# Private Interests and the Start of Fluoride-Supplemented High-Carbohydrate Nutritional Guidelines

**DOI:** 10.3390/nu14204263

**Published:** 2022-10-12

**Authors:** Philippe P. Hujoel

**Affiliations:** 1 Department of Epidemiology, School of Public Health, University of Washington, Seattle, WA 98195, USA; hujoel@uw.edu; Tel.: +1-(206)-543-2034; Fax: +1-(206)-685-4258; 2 Department of Oral Health Sciences, School of Dentistry, University of Washington, Seattle, WA 98195, USA

**Keywords:** fluoride, sugar, nutritional guidelines, dental caries, nutritional deficiencies, professional organizations, evidence-based medicine

## Abstract

Fluoride has no tangible health benefits other than preventing dental caries and there is a small difference between its minimum effective dose and its minimum toxic dose. Leading global organizations currently recommend fluoride supplementation because they recommend high-carbohydrate diets which can cause dental caries. Low-carbohydrate diets prevent dental caries making such fluoride recommendations largely unnecessary. A dental organization was among the first to initiate the public health recommendations which started fluoride-supplemented high-carbohydrate nutritional guidelines. This start required expert panels at this dental organization to reverse on three key scientific points between 1942 and 1949: (1) that topical fluoride had potential harms, (2) that dental caries was a marker for micronutrient deficiencies, and (3) that low-carbohydrate diets are to be recommended for dental caries prevention. Internal documents show that private interests motivated the events which led these expert panels to engage in pivotal scientific reversals. These private interests biased scientific processes and these reversals occurred largely in an absence of supporting evidence. It is concluded that private interests played a significant role in the start of public health endorsements of fluoride-supplemented high-carbohydrate nutritional guidelines.

## 1. Introduction

Dental caries is the quintessential disease of civilization, a disease which became prevalent with the start of cereal agriculture and rampant with the start of industrial sugar production [1]. A body of evidence supports the hypothesis that a diet leading to dental caries also leads to chronic non-communicable diseases [2]. 

Most authoritative organizations aimed to protect public health ignore this evidence and take the view that dental caries is the only adverse side-effect of their high-carbohydrate nutritional guidelines, a side-effect which can be addressed with universal fluoride recommendations. The intrinsic starches-and--sugar diet which can cause dental caries is described as healthy by the United States Department of Agriculture (USDA) [3]. The carnivorous diet which prevents and stops dental caries is described as a probable carcinogen by the World Health Organization (WHO) [4]. This latter point is raised not to suggest that the carnivorous diet is the only solution for dental caries [5], but as an example to indicate that leading organizations dismiss diets which prevent dental caries and instead rely on fluoride and food fortification to, respectively, address the dental harms and micronutrient deficiencies induced by high-carbohydrate diets.

The USDA and the WHO not only ignore the evidence that high-carbohydrate diets may lead to diseases other than dental caries, but also fail to prioritize high-quality evidence over low-quality evidence when writing their nutritional guidelines [6,7,8]. The latter observation was made by an expert who coined the term evidence-based medicine and who was a key developer of one of the most widely used evidence-based grading systems [6,7]. The USDA furthermore decided to largely ignore the 2017 recommendations of the National Academies for greater scientific rigor and thus failed to increase the trustworthiness of their scientific processes [9]. Unsurprisingly, editorials in The BMJ have described nutritional guidelines as bold policies based on fragile science [10,11].

The economic theory of public choice may explain why expert panels with a commitment to public health ignore the principles of evidence-based medicine; organizations may have biased scientific processes within their organization because of the influence of private interests, not necessarily public ones. The aim of this report is to explore the private interests which were present when a dental organization took the first significant steps towards endorsing the current fluoride-supplemented high-carbohydrate nutritional guidelines.

## 2. On the Start of High-Carbohydrate Nutritional Guidelines

“*A knowledge of the historical development of a subject is often essential for a full understanding of the present-day situation” [12]*.

Otto Warburg (1883–1970)—Nobel laureate in Physiology or Medicine—quote as described by Hans Krebs [13].

Nutritional guidelines did not move towards high-carbohydrate diets overnight. Leading professional organizations including the American Heart Association, the American Diabetes Association, and the American Dental Association all engaged in pivotal reversals towards high-carbohydrate diets over a period of decades in the 20th century. 

The American Heart Association may have been the first medical professional organization to pivot towards recommending high-carbohydrate diets. In 1957, the American Heart Association reported that there was ‘no incontrovertible evidence’ for a relationship between fat and heart disease [14]. In 1961, the American Heart Association reversed and recommended reduced fat consumption which translates on average into higher carbohydrate consumption [15]. The scientific evidence had not changed in these 4 years, naturally leading to questions on why the scientific panel appointed by the American Heart Association changed its position [16]. 

The American Diabetes Association followed later, gradually ignoring evidence on the harms of carbohydrates [17] and notably also on sucrose. In 1979, sucrose was in the category of restricted energy sources, in 1986 modest intakes were accepted for some individuals, and in 1994 the guidelines described how teens can be taught to incorporate sucrose in their nutrition [18,19,20]. The American Diabetes Association reversed its position on sucrose despite reporting that “additional research will be necessary to determine with certainty whether dietary sucrose adversely affects serum lipids”, and without evidence on whether such adverse lipid effects could translate into increased morbidity or mortality [16]. 

These medical reversals towards higher-carbohydrate diets were preceded by a dental reversal. In 1942, the American Dental Association (ADA) recommended restricting dietary carbohydrates to control dental caries [21,22]. In 1947 and 1949, this organization reversed and recommended well-balanced diets which ‘may not be accompanied by a low caries attack rate’ [23,24]. A dental organization thus appeared to be among the first to reverse away from recommending a restricted carbohydrate intake, and yet they should have been last; high-carbohydrate diets have always been regarded as harmful to teeth. 

These reversals of professional organizations are echoed in the current high-carbohydrate nutritional guidelines which are promoted by the WHO and the USDA. It has been commonly assumed that the professional organizations engaged in pivotal scientific reversals did so because of their commitment to public health. It has been commonly assumed that the expert panels which provide scientific gravitas are uniquely poised to judge a body of evidence within their specialty and can therefore reverse on guidelines in an absence of trustworthy evidence. It has also been commonly assumed that these expert panels are not inappropriately influenced by either ideology or by the authorities within the professional organization who appointed them. These assumptions can be evaluated by analyzing internal records. These records were largely inaccessible at the time of the reversals and thus led to non-transparent scientific processes. 

The internal scientific records of the dental professional organization which was among the first to endorse the start of the fluoride-supplemented high-carbohydrate diets have largely remained unexplored. These internal records provide verbatim transcripts on some of the circumstances under which experts on panels authorized key scientific reversals in an apparent absence of supporting evidence in the published scientific literature. Such internal records may offer one of the few source materials to provide transparency on the private interests which may have influenced key public health reversals. The internal records on which this report is based include the yearly bulletins of the primary scientific council of this dental organization (Council on Dental Therapeutics), minutes of the council whose primary duty was described as promoting dental public health [25], and the internal records of the Sugar Research Foundation (See Supplementary Materials, part S1 for more details on these internal records). 

## 3. A National Recommendation to Restrict Dietary Carbohydrates

*“Let us cease pretending that tooth-brushes and tooth-paste are any more important than shoe-brushes and shoe-polish. It is store food which has given us store teeth.”*

1939—Earnest A. Hooton (1887–1954) Professor of anthropology at Harvard University [26].

The ADA National Health Program Committee published a national caries control program in 1942 which was characterized by the following key points [21,22]:

- The role of bone health nutrients in dental caries prevention was described as “an established fact”.
- Restricting dietary carbohydrate intake rendered toothbrushing to be largely of social/cosmetic value.
- A toothbrush was described as an ineffective instrument in mouth hygiene.
- Fluoride was not mentioned. The key epidemiological evidence pointing to fluoride as partially inhibiting dental caries and which soon after would lead to experimental water fluoridation was largely available [27]. One of the first controlled clinical trials showing that topical fluoride applications reduced dental caries incidence was published in early 1942 [28,29] and was either not available to the writing panel or considered too preliminary. Accepted Dental Therapeutics—a yearly ADA publication authored by the ADA Council on Dental Therapeutics—cited fluoride only within the context of poisoning [30]. The ADA Council on Dental Therapeutics is henceforth referred to as the ADA CDT.

This 1942 national caries control program was approved by the ADA House of Delegates (over 300 dentists).

The 1942 national caries control program was released in an era when a group of researchers viewed dental caries as a predictor of physical degeneration. An editor of a national dental journal reported in 1945 how dental researchers had been “shouting from the house-tops” for 2 decades that carious teeth were “unmistakable sign-posts on the road that leads to” degenerative diseases, that nutrient-deficient flour and sugar led to dental caries and the “deadly touch of civilization” [31,32]. This dental editor believed that the military draft statistics for World War II had provided “irrevocable and indisputable evidence” that these dental researchers were correct; that dental caries was indeed a harbinger for the diseases of civilization. Roosevelt, the US president, had reported in 1941 that among those called for military service there was a ~20% rejection rate for dental defects and a ~50% overall rejection rate which included medical reasons such as cardiovascular disease [33]. Medical leaders, in the opinion of this dental editor had to “grasp the true implications of the situation” and act “to safeguard the health of coming generations” [31]. A book review published in a medical journal joined in the opinion that dentistry should take precedence over medicine on determining the proper nutrition to prevent the chronic diseases of civilization [32,34]. 

The view that nutritional deficiencies caused both dental and systemic diseases was not controversial. The ADA CDT reported in 1946 that “the symptoms of vitamin deficiencies often appear first and perhaps most severely in the mouth”, and that “vitamin deficiencies are likely to be associated with impairment of function in other parts of the body” [35].

Yet, ADA expert panels between 1942 and 1949 authorized 3 key reversals which may reflect the start of the public health messages on the benefits of fluoride-supplemented high-carbohydrate diets. First, the ADA CDT reversed on the safety of topical fluoride. Second, the ADA CDT reversed on the role of deficiencies in bone health nutrients as an etiology of dental caries from “an established fact” to explicitly dismissed [36]. Third, the ADA Council on Dental Health—a council committed to dental public health—reversed from the need to teach patients “that a reduction in the carbohydrate intake is necessary” to recommending a “well-balanced” diet, a term which increasingly became adopted to refer to high-carbohydrate nutritional guidelines [24]. Additional reversals which occurred concurrently at the ADA Council on Dental Health, but which are not discussed here include a 1947 first-time view that x-ray examinations are a necessary part of caries control and the 1949 reversal towards the view that a toothbrush is a public health instrument (from the 1942 view that a toothbrush was largely ineffective) [23,24]. Modern dentistry was born. 

In summary, three pivotal reversals towards the fluoride-supplemented high-carbohydrate nutritional guidelines occurred between 1942 and 1947. Expert panels with either instructions to adhere to a scientific process, or an awareness of scientific processes, reversed towards recommending nutritional guidelines which caused the disease they aimed to prevent.

## 4. A Political Leader’s Rebuke of the 1942 National Caries Control Program

*“The House ate it up. Caries Control! Boy! Just what we’ve always wanted.”* [37]

1943 David McLean—ADA Board of Trustee—reaction to the 1942 ADA national caries control program.

A key finding of this report is that public health policies can find their origin with the political leaders of an organization whose primary aims can include serving the private interest of the organization they lead, not necessarily serving the public interests. It is the political leaders of an organization who have the authority to appoint committees and its members, and this appointing authority can influence the resulting public health message.

The 1942 National Health Program Committee may have been an exception in that this panel had not been vetted by the ADA political leadership to provide public health guidelines on dental caries prevention. To recapitulate, this 1942 national caries control program focused on avoiding the micronutrient-deficient sugar and flour which were in the 1940s regarded as drivers of obesity and diabetes. Adhering to this 1942 national caries control program would have offered a glimmer of hope of mitigating our current obesity and diabetes epidemic. An ADA Board of Trustee member however did not approve of this 1942 program, reporting that it had not been authorized by the ADA Board of Trustees.

This ADA Board of Trustee reported in 1943 how the committee who wrote the 1942 national caries control program went rogue. This committee—he wrote—had been appointed to deal with a political event in Washington DC and when this political event turned into a non-issue this committee “found itself with little to do!” and studied caries control “as a stop gap” because “times were proving dull” [37].

This Board of Trustee described the conference organized by this committee as “the greatest waste of time”, and as having one presenter engaging in quackery onstage. It was a surprising accusation given the chair of the committee was a physician-dentist and past ADA president.

The view of this ADA Board of Trustee member on who has the authority to establish public health policies was noteworthy. This Board of Trustee believed major changes in public health policies needed to be studied and approved by the Board before such policy changes move to the House of Delegates for a vote. Approval of a public health policy by the House of Delegates without prior approval by the Board of Trustees was in his view meaningless—because the House is a “steam roller” affair where “the volume of business is tremendous” [38]. He reported how the 1942 national caries control program was “not read before the ADA Board of Trustees before presenting it to the ADA House of Delegates”. As a result, “a prime function of the Board (of Trustees) was nullified …that of first studying a major change of policy and passing its recommendations on to the House.” The approval by the ADA House of Delegates was in his view irrelevant; delegates were “novices” [39], selected for their ability to pay their own way to meetings [38], not because of experience in committee work. The 1942 national caries control program—in his view—thus saw the light of day because it had escaped scrutiny by the ADA Board of Trustees. The Supplementary Materials (part S2) provides an analysis on how ADA policies could become enacted in this era.

This critical assessment from an ADA Board of Trustee coincided with an almost immediate re-organization within the ADA on which panel is authorized to write public health guidelines on dental caries prevention. The 1942 national caries control program was written by the National Health Program Committee which was an independent committee. In 1942, it became a subcommittee of the new ADA Council on Dental Health—the council committed to dental public health. The first chair of the ADA Council on Dental Health had “a salaried position” as the head of the Kellogg Foundation (a cereal maker) and could therefore- in the opinion of an ADA Board of Trustee member- “give unstintingly of his time and effort” to the ADA [37,40]. This Kellogg president was not an insignificant person; he supervised 2.2 billion dollars inflation-adjusted donations from Kellogg (reported as 290 million dollars in 1970) during his subsequent 27-year tenure as president of the Kellogg Foundation. The ADA Board of Trustee member who disliked the 1942 national caries control program described one of the first presentations by this Kellogg President (and newly appointed ADA Chair of the Council on Dental Health and thus the lead person on dental public health guidelines) as “one of the finest reports the Board listened to” [41]. One year later, in 1943, in an ADA Council on Dental Health meeting, the Kellogg president commented in a fluoride-related discussion how “the thyroid problem in Michigan has been almost completely overcome by compulsory addition of iodine to salt”, to which a committee member replied “that would solve the problem (i.e., dental caries)—compulsion” [42]. It is one of the early references to the view that dental caries could be the result of a fluoride deficiency, just like thyroid problems could be the result of an iodine deficiency, and that dental caries could be solved “with compulsory addition” of fluoride to the diet. One member of the ADA Research Committee wondered whether the introduction of fluoride in the water would “initiate any diseases” to which another committee member replied: “The beneficence is sufficient to warrant the chance” [42].

Further re-organizations within the ADA Council on Dental Health led to further changes in the composition of the committee responsible for public health guidelines in nutrition. In 1944, the ADA Caries Control Committee [43] in association with the ADA Research Committee [44] became responsible for the 1947 and 1949 public health guidelines called, respectively, “The Control of Dental Caries” and “Dental Caries Prevention and Control”. 

The experts present on these 2 committees responsible for the 1947 and 1949 guidelines on dental caries prevention thus changed significantly from the 1942 committee. One initial ADA appointment to the ADA Research Committee in 1943 was Dean, one of the four pioneers in promoting water fluoridation research who would become the first director of the National Institute of Dental Research. In 1943, at the first meeting of the Research Committee of the ADA Council on Dental Health, Dean was reported as saying he believed “in the use of fluorine as a control for dental caries”, how he will not be letting “any grass grow under his feet securing convincing evidence, if he can, which will bring fluorine into general use” [42]. At this first meeting, Dean’s colleagues reported how they wanted to know all the answers about fluoride as a solution for dental caries, before “Dean sits back and says what about scurvy and pellagra”. Scurvy and pellagra are diseases caused by vitamin C and vitamin B3 deficiencies, respectively, and it was another reference to the hypothesis that dental caries became viewed by some as a fluoride deficiency disease [42]. 

The evidence on whether the ADA panels adhered to scientific rules (i.e., were unbiased) in the authorization of the key reversals are explored in Section 5 (reversal that topical fluoride had potential harms), Section 6 (reversal that dental caries was a marker for micronutrient deficiencies), and Section 7 (reversal that low-carbohydrate diets are to be recommended for dental caries prevention). 

## 5. The Start of the Fluoridated Age

*There is “an essential unity of the degenerative diseases and that dental caries is a manifestation of such degeneration…the reduction of dental caries by the fluorination of water will mask the basic problems and delay fundamental advances in public health” [45].*

1945—Paul H. Belding—Editor of “Dental Items of Interest”.

Dietary carbohydrates are a necessary cause for dental caries—few organizations apart from sugar-and-cereal-associated groups ever questioned this association. McCollum—a biochemist and discoverer of 3 vitamins—may have summarized the available scientific evidence best: only people who liberally consume dietary carbohydrates develop dental caries [46]. Fluoride lowers the dental caries risk caused by consuming dietary carbohydrates. The WHO and the USDA recommend fluoride because they recommend high-carbohydrate diets, and because they view dental caries as the only adverse consequence of such high-carbohydrate diets. One downside of recommending fluoride supplementation is its narrow therapeutic profile–a small difference between the minimum therapeutic dose and the minimum toxic dose. 

A pivotal reversal which helped to open the gates to the fluoride-supplemented high-carbohydrate dietary guidelines occurred in 1947. In 1944, 1945 and 1946, the ADA’s official policy as published yearly in the Journal of the American Dental Association (JADA) was to discourage topical fluorides because in part “the full extent of their possible harmful effects” were not known [35]. In 1947, the ADA CDT—as will now be shown—reversed on their position on fluorides in the absence of apparent new data on safety. The new position became that topical fluoride applied by dentists had “relative safety”, was effective, and could be recommended for a “highly susceptible population” [47]. It was a watershed moment for fluoride as a universal therapeutic. Soon after, the ADA Council on Dental Health recommended four separate topical fluoride applications to all children aged 3, 7, 10 and 13 years of age. Topical fluoride became endorsed at a workshop attended by a group of 100 dentists, scientists, and public health officials, by State and Territorial Health Officers, by the American Public Health Association, and by the ADA Council on Dental Health [24,48]. The ADA CDT authorized the first step to open the gates to the fluoridated age and operated under a set of adopted scientific rules. With an access to internal documents there is an ability to assess the private and public interests which influenced this reversal.

### 5.1. Fluoride Protected Sugar Markets

*“Fluorine is it.” [47]*

1945—Fice Mork—Public relation expert who was described by the First Scientific Director of the Sugar Research Foundation as “invaluable…in the matter of the dental caries problem.”

The Sugar Research Foundation was created in 1943 and funded a research agenda “to protect the existing markets for sugar and to produce new ones” [49]. 

Internal records show how the first scientific director of the Sugar Research Foundation reported that the prevalent opinion of the era was that sugar caused dental caries, obesity, diabetes, and the diseases of civilization [49]. Yet, it was dental caries which was in his opinion the disease “of great importance” [50]. He explained why. Leading authorities on diabetes—in his opinion—knew that glandular defects caused diabetes and discussions were under way with the officers of the American Diabetes Association for “suitable educational campaigns”. Medical authorities similarly knew—in his opinion—that there was no such thing as a specifically fattening food. A “Scientific Report” would provide the necessary education on obesity. The prevalent view that refined carbohydrates caused the diseases of civilization was in his view so vague as to be able to explore the opposite hypothesis—that sugar protected against the diseases of civilization [49]. 

The first scientific director of the Sugar Research Foundation believed that educational campaigns would not solve the dental caries problem. Internal documents show how this scientific director had discussed the “entire question” on how to solve the dental caries problem “for hours at a time” with the public relation expert who had first worked for the ADA and who now was working for the Sugar Research Foundation [51]. Educational campaigns on oral hygiene in these discussions were viewed as useless largely as a result of a dental caries conference held in New York in 1934 [52]. The consensus opinion of this conference—as headlined in the New York Times—was that oral hygiene was useless [52]. The public relation consultant reported in 1945 how sugar interests would “do all right” if they searched for the facts on fluoride [51]. Already in January 1944, the first scientific director of the Sugar Research Foundation had predicted in one of his first public addresses a coming “carbohydrate age” and how dietary fluoride needed to be taken in adequate quantities as part of this diet [50]. This 1944 public address was sent to all US college and university presidents [53]. The first scientific director thus predicted a carbohydrate age with fluoride supplements which is subsequently referred to here as the fluoridated carbohydrate age.

Three examples show how the Sugar Research Foundation and possibly others may have promoted this fluoride message.

First, the Sugar Research Foundation funded a conference on water fluoridation which was held on 30 October 1944 and the aforementioned public relation expert had a hand in appointing at least one speaker who opined that “sugar is not a cause of dental caries” [54,55]. As a “public service”, the Sugar Research Foundation paid for the printing and mailing of the conference proceedings to all practicing dentists in the United States, to members of the American Academy of Pediatrics, and to the senior dental class at universities [56,57]. A past ADA president prefaced the sugar-funded conference by suggesting that water fluoridation offered the possibility of “immunizing an entire community against dental caries”. 

The public relation expert working for the Sugar Research Foundation reported on the impact of mailing out the conference proceedings as follows: “Following the distribution of this book on fluorine, many local health departments have started agitating for a fluorine program of their own. There is no doubt in my mind that there is a close connection between the distribution of the booklet on fluorine by the New York Institute of Oral Pathology and this agitation.” [57] A fluoride pioneer reported later how that meeting “will stand out in the annals of American dentistry because it helped to focus attention on a new public health procedure which promises to be a milestone in modern public health” [58].

Second, even before this conference, public demand for fluoride was created by newspaper articles. A state organization of a pharmaceutical group had reported how a local newspaper article on fluoride preventing dental caries had led to 200 to 250 calls by laymen to pharmacists. Some of these pharmacists then sold fluoride to consumers “thinking they were going to use it as rat poison”, and, yet lay people bought it to prevent dental caries. The medical director of the Food and Drug Administration (FDA) reported how accidental deaths were “very definitely” a possibility [59].

A dental editor reported in 1945 how these “untimely blasts of lay publicity” on fluoride” so out of proportion to established scientific facts, have made their appearance as to lead to the belief that they might be fostered by an ulterior motive” [45]. He reported how a congressional investigation would be highly desirable. 

Third, the ADA Council on Dental Health reported in 1946 how they had “recently seen some literature to be sent to the public urging them to question their dentists (on fluorides) [60]”. Probably because of all this publicity, the ADA Council on Dental Health reported in 1946 “receiving a large number of inquiries about the use of fluorides as a public health measure for preventing dental caries”. The ADA CDT—which still discouraged fluoride use in 1946—was informed by the ADA Council on Dental Health on this last point.

### 5.2. Fluoride, Dental Ideology, and the Fifth Principle Which Was Dental Caries Prevention

*“It’s this kind of procrastination and fence straddling on the part of organized dentistry that will bring on the socialization of our profession quicker than all the communists put together”.*

1945—J.G. Frisch—dentist.

Fear of socialized medicine was another private interest pushing towards the acceptance of fluoride as a dental therapeutic and thus possibly influencing the ADA CDT. The dental profession was perceived as failing to provide dental care for 80% of the US population in the late 1930s. Dental caries was rampant. Politicians in Washington DC aimed to create socialized medicine to improve the health of the nation. The ADA had not been officially invited in 1938 to participate in this first National Health Conference. This was the start of an existential crisis for the dental profession. The fear was present among some ADA leaders that the dental profession could become integrated into the medical profession as a workforce that drills and fills teeth and that makes dentures. 

The survival of an independent dental health profession was uncertain in the early 1940s. The American Medical Association (AMA) was described by the chair of the ADA Council on Dental Health as viewing dentists, optometrists, osteopaths, and chiropractors as minority groups [61]. “Medicine has swept plenty of dirt under its carpets” he reported [61]. Some ADA leaders perceived dentistry as being at risk of being swept up under the carpet. “We are just like the optometrists!” exclaimed an ADA Board of Trustee expressing his dismay at how a University President had compared dentists to optometrists, and physician-dentists to ophthalmologists [62]. 

Some editorials in dental journals of this era perceived adverse consequences if the dental profession became absorbed into the medical profession. An ADA Board of Trustee wrote how the US president (and the US President’s wife) looked favorably upon a plan to make dental patients roll down production lines. With this plan, dentists would drill and fill on the belt line. Physicians would supervise the belt line; diagnose and treatment plan. “Droves of sublevel dentists” (repair man or tooth carpenters) would be “supervised by super-dentists integrated into Medicine” [62]. There was a fear that the medical profession in a “socialized medicine setting” would be holding the purse strings for dental care, that the medical profession would integrate dental education into medical education. 

Medical imperialism, according to an editorial, “had to be destroyed once and for all time” [63]. ADA accreditation of a dental school which became incorporated in a medical school was swiftly suspended [64,65,66]. An ADA lobbying office was opened in Washington DC in 1943 in part to fight off such medical dominance over the dental profession [41,67]. Lobbying for independence bore fruit. Within a year, this investment in a lobbying office in Washington DC paid “more dividends to the dental profession than any other recent investment” [68]. 

Destroying this medical imperialism—as will be shown later—ended up implying to some leading dentists that existing medical science on dental disease management should be destroyed and that a new dental science should be created. A 1944 article written by a dentist referred to the need of finding an “ideological key” which would make dentistry equal to medicine [69]. This dentist reported that this ideological key was “dental science-knowledge” or dental “biological sciences” which he described as being in an infantile stage in 1944 [69]. 

A mission of dental disease prevention had been given as one of the reasons why the ADA needed to be recognized as an equal to the AMA in the formulation of any socialized medicine plan. The ADA National Health Program Committee went to Washington DC in 1939 to present to a US Senate subcommittee a list of 8 such principles to recognize the ADA as equal to the AMA. The fifth ADA principle stated that “the dental phase of a national health program should be approached on a basis of prevention of dental disease” [70]. 

Fluoride ended up fitting the bill in fulfilling both the promise of the fifth principle and the adherence to the ideological key for dentistry to be independent of medicine. Research on the effectiveness of fluoride for dental caries prevention had been mostly performed by dentists, not physicians. One leading dental actor in the fluoridation debate [71] believed that the evidence in support of fluoride had been available since 1937 and that failure of the ADA to accept fluoride as a primary prevention tool would lead to socialized dentistry. This enthusiastic promotor of fluoride reported that the ADA Research Committee (which in part worked on water fluoridation) was made up of “a bunch of schoolteachers that never made a denture in their lives, spends 52 bucks out of $16,000 for dental caries research and they are the group who say we do not know enough about fluorine” [72]. He perceived that the public demand for fluoride created by lay publicity was being stonewalled by the ADA by “some downright falsehoods”, by “desperately trying to give (fluoride) a black eye” [73]. 

### 5.3. The Political ADA Reversal on Fluoride and Dental Caries

*A JADA editor “in the pillory prior to being drawn and quartered”.*

1944—Editorial in Items of Dental Interest [74] on the demise of a JADA editor.

As stated earlier, a key finding of this report is that reversals in public health policies can find their origin with the political leaders of an organization whose primary aim may be to serve the private interest of the organization they lead, not necessarily public interests. The ADA reversal on topical fluoride safety may be no exception in this regard. Political leaders in this instance were accused of politicizing the central office of the ADA organization. A dental editorial warned how “internal strife will dog the footsteps of the (American Dental) Association as long as political factions attempt to control the central office of the ADA through political appointments” [75]. A particular bone of contention was the appointment of a new JADA editor in 1945 which “rumor has it” was decided by “a majority group of the Board of Trustees of the ADA…with little consideration of the wishes of the dental profession” [75].

This transition process to a new JADA editor may have started as early as 1943 and was described as “one of the most diabolical and undercover campaigns imaginable” [76], how there was “extreme delight in crucifying the reputation of brilliant men” [76], how this became “such a dark blot on the escutcheon of the ADA that it could well be the subject matter for a long sermon” [77]. In February 1944, the ADA Board of Trustees decided unanimously to let the contract of the 1944 JADA editor expire [67]. This editor resigned and did so “with regret” [78]. 

This JADA editor who regretfully resigned was described in Sugar Industry correspondence as one of the ADA officials with a prejudice against sugar, who had told them “bluntly that he would not publish any information about the Sugar Research Foundation” [55]. 

This JADA editor also had concerns about fluoride safety. He authorized the publication of an editorial which appeared on 1 October 1944 in JADA and which stated that “we do know that the use of drinking water containing as little as 1.2 to 3.0 parts per million of fluorine will cause such developmental disturbances in bones as osteosclerosis, spondylosis and osteopetrosis, as well as goiter“ [79]. This editorial stated that “the potentialities (of fluoride) for harm far outweigh those for good”. This JADA editorial had the opposite public health message of the Sugar Funded conference on fluoride which was going to be held 4 weeks later—on 30 October 1944.

Three events followed the publication of this JADA editorial on the harms of fluoride.

First, the scientific director of the Sugar Research Foundation met with key ADA officials at the ADA convention in Chicago (16–18 October 1944) to set up a dental advisory committee. The “officials of the ADA agreed to cooperate” [56]. 

Second, at this same convention in Chicago, the ADA Board of Trustees proposed a new JADA editor which one editorial described as being “heartily disapproved by great numbers of the ADA” [75]. It was reported this new appointment could only succeed if the ADA Board of Trustees was able to pull the wool “over the eyes of the House of Delegates” [74]. The ADA Board of Trustees did succeed in this task. 

Third, this new JADA editor appointed by the ADA Board of Trustees met with representatives of the Sugar Research Foundation within 2 months of the start of his appointment. The first scientific director of the Sugar Research Foundation was described as “very pleased about the results of this meeting” and that the meeting was “completely successful”. This new JADA editor agreed to “carry announcements about future Sugar Research Foundation grants”, to “carry news announcements”, and he “even offered to act unofficially and make suggestions (to the first scientific director of the Sugar Research Foundation) from time to time about the standing of various individuals in the profession” [55]. 

These events relate to the reversal of the ADA CDT on the safety of fluoride. In 1946, the ADA Central Office (where this new editor resided) invited the ADA CDT to review and accept topical fluoride products for use in the dental office. The ADA CDT refused [60]. In 1947, the new JADA editor took “on his own responsibility” the decision to publish an editorial on the safety (and efficacy) of topical fluoride. The new JADA editor had decided—after discussions in the ADA Central Office—“that the scientific evidence to support application of fluoride in the dental office by dentists had reached a stage where this procedure could be encouraged and recommended”. The editorial he authorized stated that the toxic potentialities of fluoride were “not significant” [80]. The ADA CDT at this point still had the position that the “full extent of their possible harmful effects were not known” [35]. In the same year, this same JADA editor authorized the publication of an editorial on the need for further studies on the effectiveness of water fluoridation, not mentioning the need to study the specific harms of fluoride which were reported as known in the prior JADA editorial [81]. 

It is not implied here that sugar interests necessarily had a hand in this transition process at the ADA Central Office. It is possible that the events at the ADA Central Office described in this section are unrelated to sugar interests and any such appearance may be the result of coincidences. At least one ADA Board of Trustee did not bring up sugar interests when explaining why the sugar-hostile JADA editor was let go [67]. Neither is it implied here that the authority of the ADA Board of Trustees to appoint key ADA officials within the central office of their organization necessarily implies that scientific panels within their organization will forego their scientific rules. The next sections explore whether scientific processes have the potential to become biased because of what at least some leaders in the dental profession regarded as the politicization of the ADA Central Office.

### 5.4. On Public Health and the Narrow Therapeutic Index of Fluoride

*“At the present time the fluorides are our number 1 problem and the reason we regard them as such is the possible toxicity of these preparations” [82].*

1947—R.F. Stormont, Acting FDA Medical Director.

The ADA CDT had yearly in person-meetings which were often attended by an FDA official. Internal records suggests that the presence of this FDA official may have been one reason why the ADA CDT published warnings on the potential systemic harms of fluorides from 1944 until 1946.

In 1944, the FDA Medical Director informed the ADA CDT that they were receiving 5 inquiries a week for the desirability of putting fluoride products on the market. These products included systemic fluoride treatments (e.g., bone meal with fluoride) and topical fluoride treatments (e.g., toothpastes, rinses, and pastes). The FDA director stated that the key public health problem for fluoride was ingestion of a “protoplasmic poison [59]”. One opinion of the era appeared to be that topical fluorides—when not ingested—were safe. The FDA Medical Director, for instance, stated: “they say it is perfectly safe to put fluoride in a mouth wash, for example, and permit it to be sold on the market [59].” One can assume that the “they” the FDA director referred to were representatives of the companies submitting topical fluoride products to the FDA. The FDA director did not share this opinion because of concerns related to accidental swallowing of topical fluoride products.

In 1944, the FDA Medical Director furthermore reported how the safety of applying fluoride to teeth presents “a very serious problem primarily because the difference between the amount which may have some benefit, and that which will definitely be toxic, has a very narrow range” [83]. New drug applications for fluoride needed to “convince any reasonable scientist that this particular product under the proposed conditions of use would be safe for use [59].” The response from industry was reported to be a constant: “Obviously we can’t get such information at this time [59].”

In 1945, the FDA Medical Director reported on a hearing where there was “sufficient testimony “that fluoride and many of its compounds “were poisonous and deleterious substances” [83], and “that a sizable proportion of the (US) population is exposed to toxic or near toxic quantities of fluorine and any addition will only increase the toxicity resulting from fluoride intake” [83]. The main concern reported by the FDA medical director was the ingestion of fluoride pesticides and fluoride-containing foods in the US population. Dental fluoride products would increase such fluoride exposures. Effectiveness of fluoride as a dental caries prevention method was not reported to be the issue. The FDA medical director reported that “there has been adequate work to show that you can reduce the incidence of caries in children (with fluoride) [83]”.

In 1946, the FDA was represented by an AMA official, and the discussion focused largely on fluorides in toothpastes and desensitizers. In 1947, the FDA acting medical director reported how fluorides (among dental products) were “their number 1 problem” in the dental arena and the reason the FDA regarded them as such was “the possible toxicity of these preparations” [82]. The next section shows how the ADA CDT reversed on the safety of topical fluorides soon after having listened to this message of the 1947 FDA director. 

### 5.5. The Public Health Endorsement of the Start of the Fluoridated Age

*“I think it is a bad policy. I can see where it (i.e., the reversal on fluoride) might embarrass the Food and Drug Administration” [82].*

1947—Harry Lyons—ADA CDT member and future ADA president.

From the perspective of the published scientific literature, the ADA CDT’s 1947 approval of the effectiveness of topical fluoride can be interpreted as the logical consequence of the accumulating evidence of fluoride’s effectiveness in dental caries prevention as demonstrated in controlled clinical trials. In 1945 already an ADA CDT member reported: “It is a pretty well accepted fact that you can decrease the incidence of dental caries in children (with fluoride)” [83]. 

From the perspective of the published scientific literature, the ADA CDT’s 1947 reversal on the safety of topical fluoride is challenging to explain. The ADA CDT’s policy statements from 1944 to 1946 were driven in part by safety concerns. The Chair of the ADA CDT reported in 1944: “You really have got to protect the public not only from the things they buy on the market, but from certain other individuals who practice medicine and dentistry who will pass these (fluoride products) along [59].” 

This section focuses on the reversal of the ADA CDT with respect to the harms of topical fluorides as applied by dentists. The ADA CDT published in JADA in 1944 and 1945 that the full extent of the possible harmful effects of fluorides were not known [84,85]. The FDA medical director reported in 1945 to the ADA CDT how such ADA CDT public statements on the non-acceptance of fluoride products for dental caries prevention were a “very substantial help to” the FDA, how “particularly the hazard part” of fluoride was helpful [83]. 

Whether the concern on the potential harms of topical fluoride applications extended to in-dental-office use (as opposed to over-the-counter fluoride products) can be viewed as ambiguous in the first official ADA policy statements in 1944 and 1945 [84,85]. One ADA CDT member reported how the actual first statement was “so complicated” … “that unless the dentist is a lawyer you won’t be able to interpret it” [59]. 

Both internal and published statements show no such ambiguity on the type of topical fluoride products for which ADA CDT listed safety concerns in 1946. In 1946, the ADA CDT explicitly referred to concerns in approving topical fluorides as applied in the dental office. The ADA CDT Secretary had asked the ADA CDT in 1946 to consider standards for topical fluoride products for use in the dental office. The lead ADA CDT writer on ADA fluoride policy replied: “The answer is no… There is no evidence of any significance in the past year to modify the position of the Council that is so well expressed in the report of the last meeting” (i.e., the 1945 statement published in JADA which included concerns on safety) [60]. The ADA CDT Secretary appeared to agree that he was mistaken to ask for approval of topical fluoride brands for in-office use. He reported: “When this agenda was compiled I did not have access to (the lead ADA CDT fluoride expert’s) latest publication on topical application of fluoride which makes it appear now that acceptance of those products would be premature” [60]. The ADA CDT’s published statement appeared also explicit in this regard. The ADA CDT stated in the 1946 JADA report that topical fluorides should not be used in “routine dental practice” because the “full extent of their possible harmful effects are not known” [35]. 

The ADA CDT reversed on this safety statement in 1947 in an apparent absence of evidence. Internal records show that the ADA CDT became informed at their annual in-person meeting in 1947 that the ADA Central Office had made the decision to publish an editorial that topical fluorides as applied by dentists were safe and effective without prior scientific review by the ADA CDT. The ADA CDT was informed that this decision was made because “the feeling (in the ADA Central Office) was so strong that this (i.e., topical fluoride) was a good thing and it should be recommended to the profession [82].” This decision was made by the new 1945 JADA editor with sugar connections who “was willing to make that recommendation on his own responsibility” [82]. It offers a first example where the scientific processes of the ADA CDT had the potential to become biased when a pivotal reversal on a public health issue was in the making.

The ADA CDT agreed to reverse from their 1946 statement that the “full extent of their (i.e., topical fluorides for in-office use) possible harmful effects are not known”, to what would become their 1947 statement that topical fluorides as applied in the dental office had “relative safety [82]”. 

The ADA CDT expressed several opinions which may have led them to reverse their position independently of the pressure created by the JADA editorial. One reason for agreeing to reverse may have been that their fluoride safety warnings had always been driven by a desire to help the FDA in keeping topical fluoride products out of the hands of consumers (i.e., a commitment to the greater public good), not by a concern on the safety of topical fluorides as applied by dentists. The following sentence, written by a fluoride pioneer, was read to the ADA CDT in their 1946 in-person meeting: “We are not impressed by any harm that might follow the dentists careful use of fluorides in topical application but we would stress the fact that they are poisonous and so are not safe agents to put in the hands of the public [60].” The ADA CDT did not raise an objection to this statement possibly suggesting they were similarly not impressed by harm. Another reason for the ADA CDT to agree with the reversal may have been the decision (by an undisclosed party) to invite Knutson—a prominent clinical trialist on topical fluorides—to give a presentation to the ADA CDT on the controlled clinical trial evidence to date. Knutson’s track record on published fluoride research was more extensive than the lead ADA CDT fluoride expert. This presentation on 7 March 1947 offered a systematic review of the evidence on effectiveness, not safety, and included a large, controlled trial which Knutson had conducted and which the ADA CDT members likely had not yet seen. (This trial became published 2 weeks after Knutson’s in-person presentation to the ADA CDT on 21 March 1947) [82]. Still another reason may be that the reversal on safety of topical fluoride was limited to a “highly susceptible population” and did not extend to statements that over-the-counter topical fluoride products were safe. One ADA CDT member reported after the reversal: “This (approval) in no way alters the rest of the Council’s stand on fluorides” [82]. The reader should be reminded here that all these plausible reasons are mostly available from internal documents. Any interested party looking at understanding the 1947 reversal of the ADA CDT on the safety of topical fluoride from the perspective of the published evidence would be faced with a challenging task.

As an aside, one study (on a single subject) on the safety of 0.4% topical fluoride applications appeared not to have been discussed at the ADA CDT meetings between 1944 and 1947 [86].

No judgment is made here whether the ADA CDT safety statement on topical in-office fluoride use was correct in 1946 or in 1947. The main point here is that the 1947 reversal on the safety of fluoride is largely inexplicable from a scientific perspective as the evidence on safety had not changed. Internal records indicate that no evidence on the safety of topical fluoride, which could have justified a reversal, was discussed. To a large extent, all this lack of evidence should not come as a surprise. The ADA CDT itself indicated in 1944 and 1945 that they were not operating under their adopted scientific rules by prefacing the JADA fluoride statements with the following pre-amble: “the ADA CDT is of the opinion that…” [84,85]. 

The main point here is that there was no scientific basis to justify a reversal on safety and that private interests had the potential to bias this reversal. As one ADA CDT member noted, this reversal was initiated with “bad policy” on the part of ADA Central Office and would have created “an embarrassing conflict” if the ADA CDT had stuck with their 1946 position and thus taken “a contrary stand” to what had been decided in the ADA Central Office [82]. It was a small step for the ADA Council on Dental Health to extend soon after the topical fluoride indication from a “highly susceptible population” to repeated applications regardless of dental caries susceptibility. It was the start of a perceived universal fluoride need. 

## 6. The Start of a Potential Micronutrient-Deficient Age

*“The Mouth is frequently a sensitive indicator of nutritional maladjustment. The teeth may be the first to betray abnormalities in the whole skeletal structure and the gums may reveal deficiencies which exist in other parts of the body as well* *” [82].*

1943—Robert S. Harris—nutritional biochemist and professor at MIT [87]. This quote appeared in an appeal to the ADA Council on Dental Health to join other organizations in nutrition research.

The current conventional wisdom on pediatric dental caries prevention is that teeth should be mineralized and re-mineralized with fluoride, not with providing an adequate intake of vitamin D and minerals which have the additional benefit of preventing a life course leading to osteoporosis. 

The pivotal ADA CDT reversal explicitly dismissing the role of micronutrient deficiencies in dental caries etiology occurred in 1945. This ADA CDT reversal may have in a small way contributed to turning the carbohydrate age (which the Sugar Research Foundation had predicted) into a micronutrient-deficient carbohydrate age. This reversal was not influenced by the Sugar Research Foundation. The First Director of the Sugar Research Foundation was concerned about micronutrient deficiencies induced by carbohydrate-based diets and interested in food fortification if research showed it was needed. The AMA, the FDA, leading nutritionists, the Sugar Research Foundation, and the ADA in its official policy had all expressed the need to address micronutrient deficiencies in the US population. 

The 1942 national caries control program stated that it was “an established fact” that bone health micronutrients “retard the progress of caries of the dentin and in some cases causes its complete arrest” [22]. In 1945, the ADA CDT authorized rejecting this “established fact”, and in 1949, the ADA Council on Dental Health stated that bone health micronutrients have “not been shown to have any relation to dental caries” [24]. 

The ADA CDT authorized this reversal and, in this instance, showed an apparent disregard for the positive contributions dental research could make to the greater public good. The late 1940s was a critical era in shaping the micronutrient density of the coming carbohydrate age. Nutritionally enriched carbohydrate foods such as vitamin B1 fortified flours were about to enter the marketplace. The FDA had the general position not to adopt any regulations against food fortification due to its perceived importance for public health [88]. The ADA CDT had been informed that the FDA and the AMA aimed to address nutritional deficiencies in the US population, and yet were about to authorize a reversal which would mark the start of ignoring dental symptoms as one of the most sensitive signs for specific micronutrient deficiencies.

The ADA CDT reversal on vitamin D—a bone health micronutrient—may have contributed to a failure to safeguard the bone health of 3 future US generations. Clinical research with dental caries as the endpoint had suggested in 1941 that the daily vitamin D requirement needed to be increased by 50% (from 400 to 600 IU) [89]. This estimated higher requirement turned out to be correct. The AMA reported it would re-consider the request by milk manufacturing companies to allow this claim if more conclusive evidence became available [89]. The ADA CDT however may have helped to stop such further considerations by stating that vitamin D played no role in dental caries prevention. It took the Institute of Medicine another 70 years to conclude that the daily vitamin D requirement was indeed 600 IU [90]. 

Hypovitaminosis C as a marker for a gingival bleeding tendency became similarly dismissed by the dental profession, and as a result the World Health Organization may still underestimate the human vitamin C requirement [91]. 

The potential role of other micronutrients in dental disease prevention—an active research area prior to the reversal on vitamin D dental caries prophylaxis—also became largely ignored. This potential negative dental contribution started with the vitamin D reversal announcement which was authorized by the ADA CDT in 1945 [92].

The events which led to the dismissal of vitamin D dental caries prophylaxis also started with ADA officials—not the ADA CDT. In a prior section of this report, it was pointed out how a member of the Board of Trustees believed public health policies need to be approved by the Board of Trustees. In the next section it is shown how a Board of Trustee member with ‘a good head on his shoulder, and both feet on the ground” [41] may have had a hand in a major change in an ADA public health policy. Once again, a pivotal public health reversal appears to have started with political leaders.

### 6.1. Miracle Toothbrush Claims Made a Dental Organization’s Hair Stand on End

*“America’s Health is a passport to Victory. Guard it by spreading the gospel of oral hygiene, to prevent health imperiling tooth decay. Recommend that your patients use Dr. West’s Miracle-Tuft Toothbrush as least twice daily.”*

1943—Toothbrush advertising claims as published in the Journal of the American Medical Association (JAMA) during World War II [93].

The ADA financial balance sheet was mostly in the black during World War II. Income from exhibitors during national meetings was down [94] but was offset by increases in membership [95], revenue from good investments [96], and increases in JADA advertising revenues. First quarter ADA advertising revenues increased slightly from 1942 to 1943 [96], and by 1945, the year of the ADA CDT reversal on the role of bone health nutrients, advertising revenues had increased a 100% since 1943 [83].

**Figure 1:**
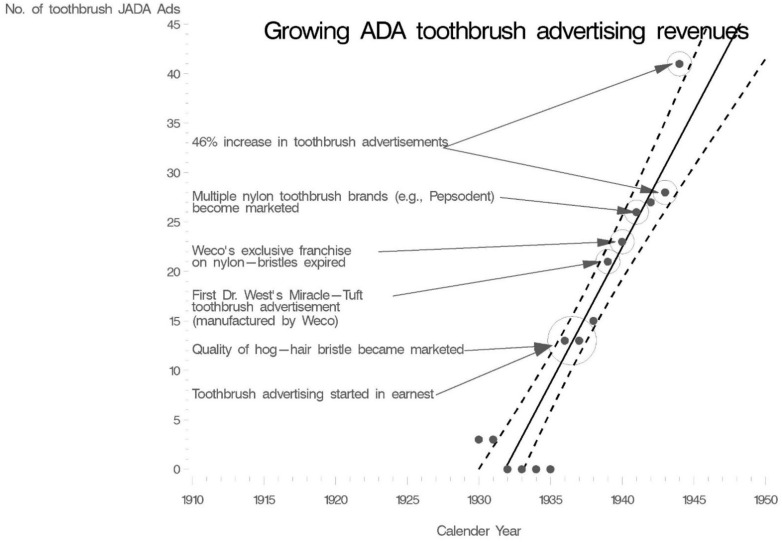
Toothbrush advertisements were a growing source of ADA advertising revenues. In 1942, the National Health Program Committee had reported a toothbrush was largely ineffective in dental caries control. In 1949, the ADA Council on Dental Health reported that “it is now believed that brushing of the teeth” is of value in dental caries control [24].

This growth in JADA advertising revenues was in part driven by increases in toothbrush advertisements (Figure 1). Dr. West’s Miracle-Tuft toothbrush was the first nylon toothbrush on the market in 1937. In 1940, the exclusive franchise which allowed Weco^®^, the manufacturer of Dr. West’s Miracle-Tuft toothbrush, to have exclusivity on nylon-bristled toothbrush production expired. Other companies started to market their branded nylon-tufted toothbrush designs. This created a surge in advertising. Pepsodent^®^, for instance, introduced its first nylon toothbrush in 1941 and spent 21 million dollars (inflation-adjusted) on radio spots, color advertisements in weekly magazines, newspaper supplements, and JADA advertisements [97,98,99]. By 1944 there were 6 toothbrush manufacturers advertising in JADA. These companies were advertising heavily and vying for a share of the half-a-billion dollars (inflation-adjusted) that Americans would spend on toothbrushes in 1944 [100]. In 1944, Dr. West’s Miracle-Tuft toothbrush was still a market leader outselling other toothbrush brands 2 to 1 [101]. 

**Figure 2:**
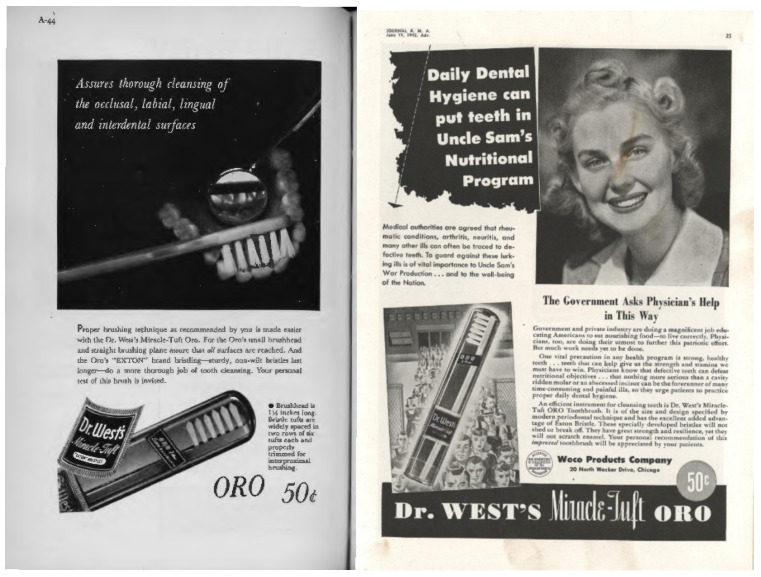
Advertisements for the first nylon-tufted toothbrush in JADA (**left**) [102] and JAMA (**right**) [103]. The ADA allowed toothbrushes to be advertised as cosmetic tools; the AMA authorized toothbrushes to be advertised as public health instruments. These differences in allowable advertising claims created an ADA-AMA conflict.

In 1943, JADA carried advertisements for Dr. West’s Miracle-Tuft toothbrush without therapeutic claims and without an ADA endorsement seal (Figure 2). The absence of an ADA Seal was logical; devices were outside of the scope of authority of the ADA CDT in 1943. Hence, the ADA CDT had not decided whether a toothbrush could advertise a dental caries prevention claim in JADA and whether it could carry the ADA Seal in advertising. JAMA carried advertisements for the same toothbrush with dental caries prevention claims (oral hygiene prevented dental ‘decay which may lead to neuritis, arthritis and other maladies’) and with an AMA endorsement seal (“Accepted for advertising in publications of the American Medical Association”) (Figure 2). Executives of the company with the market-leading toothbrush preferred advertising in JAMA which allowed therapeutic claims. In 1943, JAMA had 17 Dr. West’s Miracle-Tuft toothbrush advertisements; JADA had 2 such advertisements. In a strange twist of events, these AMA-ADA differences in allowable claims in dental advertisements would lead to the dismissal of the vitamin D dental caries prophylaxis claim.

### 6.2. The Political Reversal on Micronutrient-Deficiencies and Dental Caries

*“Dr. Washburn has shown a very intensive interest in our affairs…” [104].*

1943—Harold S. Smith, Chair of the ADA CDT from 1932 until 1947. He introduced Harvey B. Washburn—ADA Board of Trustee member—at the ADA CDT meeting on the eve of conflicts with the AMA.

It is unclear who at the ADA became first aware of the JAMA advertisements for Dr. West’s toothbrush. Washburn—an ADA Board of Trustee member—asked and received in 1944 the power to act on behalf of the ADA CDT in matters related to advertising [59]. It is a second example where the scientific rules adopted by a scientific council became ignored, and which allowed a political figure (unclear whom) to take care of a private interest—conflicts on advertising policies with the medical profession. Within a month of the ADA Board of Trustee’s request, the ADA Central Office–which falls under the authority of the ADA Board of Trustees—started to negotiate with the AMA on their advertising policies for products of potential dental significance. While initial ADA Central Office demands in this conflict were modest, they ultimately escalated towards suggesting ADA control over allowable dental advertising claims in AMA journals [105]. The AMA refused. This failure of the ADA Central Office to come to an agreement with the AMA led to an involvement of the ADA CDT in the conflict—to add scientific gravitas to ADA Central Office demands. The involvement of the ADA CDT failed to change the AMA’s position.

The aim of the ADA Central Office, as described later by the ADA CDT’s Secretary [106], was to now impress the AMA that the knowledge of the ADA CDT on dental issues was “so definite and extensive” that the ADA did not need to make an apologetic stance towards the AMA in terms of determining allowable dental claims in advertisements. An ADA CDT referee was asked to write a report on a JAMA advertisement with a vitamin D dental caries prophylaxis claim [36]. This report was critical of the way a vitamin D claim was advertised in JAMA and was sent to the AMA for comment. The AMA did not agree with the ADA referee report. The next step in the conflict was the ghost-written announcement in JADA stating that vitamin D did not offer effective dental caries control.

An ADA official wrote the reversal announcement on vitamin D dental caries prophylaxis [36]. The ADA central office aimed to publicize that the AMA had no scientific authority on dental disease prevention claims (without mentioning the AMA by name and without referring to the AMA advertisement under dispute [36]).

It was ADA officials who initiated a conflict on the advertising policies with the AMA, who requested a ghost-written reversal to be written on vitamin D dental caries prophylaxis after negotiation efforts failed, and who picked the ADA CDT to authorize this ghost-written statement. These three decisions were logical from a non-evidence-based perspective. 

**Figure 3:**
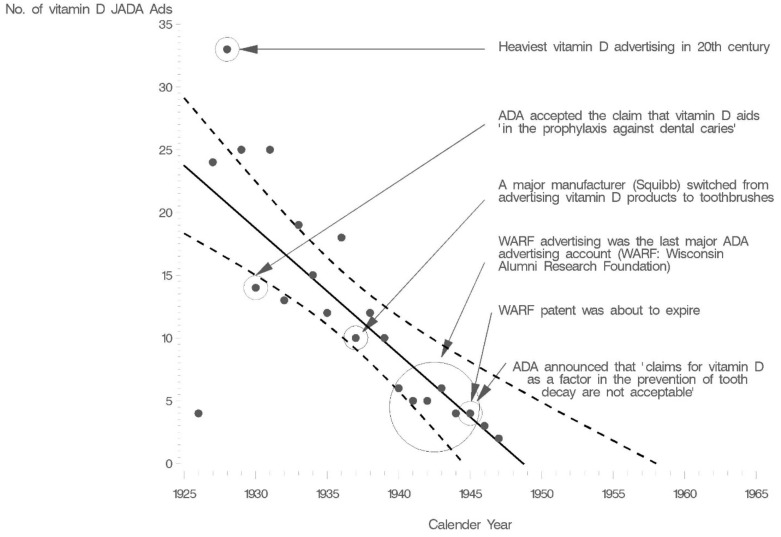
The ADA endorsed vitamin D dental caries prophylaxis in 1930 when vitamin D advertising revenues were high and when the Medical Research Council in the United Kingdom (UK) still considered the evidence untrustworthy. This endorsement reversed in 1945 when ADA vitamin D advertising revenues were about to go extinct but when the US National Research Council started to consider the evidence trustworthy [36].

First, bone health nutrients such as vitamin D, regardless of their public health importance, had ceased to be of economic value to the ADA. The ADA advertising revenues for vitamin D products were about to become zero due to the imminent expiration of key industrial patents for vitamin D production (Figure 3). ADA officials also did not have to worry about their announcement causing an income loss to ADA members. Dentists’ revenues were derived from dental procedures, and likely hurt by giving nutritional advice (more on that later). The new chair of the Caries Control Committee a subcommittee of the ADA Council on Dental Health) had published an article in 1943 stating that “recommending vitamins and minerals as caries preventives is an ideal way to ruin a dental practice” [107]. 

Second, from a policy perspective—not from an evidence-based perspective—the ADA Central Office had a valid point in contacting the AMA for advertising unacceptable dental claims in AMA journals. The AMA had not officially accepted a vitamin D dental caries prophylaxis claim (only the ADA had done so). Thus, the AMA was endorsing vitamin D advertising claims in JAMA which their own AMA councils had not yet approved. Furthermore, the ADA Central Office had a recent AMA referee report expressing skepticism on vitamin D dental caries prophylaxis claims. This may have suggested to ADA officials (mistakenly so) that the AMA Council on Nutrition was not in the process of reversing towards allowing vitamin D dental caries prophylaxis claims in advertising. 

Finally, once again, the key officials at the ADA Central Office can make an educated guess on which Council or Committee in their organization may be inclined to underwrite a key scientific reversal for the organization. For the vitamin D reversal, the ADA CDT’s secretary reported how the ghost-written announcement recorded the views of the ADA CDT in a publicly accessible form. The secretary was anticipating, just like for the fluoride reversal, that the Council would authorize the reversal [105].

Internal records explain where this expectation of the Secretary of the ADA CDT found its origin. The ADA CDT lead vitamin expert was, during his 17-year tenure, opposed to the dental profession endorsing pharmaceuticals which he considered as falling into the medical scope of practice [60]. He agreed with dentists recommending oranges and cod liver oil (household products), not with dentists recommending pharmaceutical vitamin C and D products. He approved of the ADA CDT endorsement of cod liver oil, but was opposed to ADA CDT endorsement of vitamin D pharmaceuticals (vitamin D2 and vitamin D concentrates) in 1930 (in part because of his scope of practice concerns) and he remained opposed to ADA CDT endorsement of such vitamin D pharmaceuticals (which fell in the medical scope of practice) until 1946 [60]. He was overruled on his opinion that the ADA should not endorse such pharmaceutical vitamin D products in 1930 [60]. The ADA CDT may have never endorsed vitamin D dental caries prophylaxis with pharmaceutical vitamin D products were it not for this overruling of the ADA CDT lead vitamin expert.

In addition, some dentist members of the ADA CDT were skeptical of accepting the medical management of dental caries already in 1930 for an aforementioned reason, dental ideology. Dental ADA CDT members objected to the medical management of dental diseases. One dental ADA CDT member reported in 1930 that he found it “somewhat objectionable” that the treatment of dental caries requires “accurate diagnosis (of nutritional deficiencies) which is the duty of the physician” [108]. Another dental ADA CDT member reported in 1943, “for the record”, that several consultants for the National Health Program Committee were “strongly opposed” to the publication that it was “an established fact” that bone health nutrients play a role in dental caries prevention [22,30]. Fluoride pioneers such as Trendley Dean were skeptical of nutrition’s role in dental caries etiology in general [109] and at the ADA Research Committee he “saw no reason for the committee” in approving a vitamin D research project on dental caries prevention [82].

The Secretary of the ADA CDT, because of his knowledge of these past attitudes of ADA CDT members, anticipated that the ADA CDT would authorize the ADA reversal on vitamin D dental caries prophylaxis. He may have been not entirely accurate in his anticipation. The proposed public service messages “Vitamin D—not effective against dental caries” were not adopted by the ADA CDT and thus did not see the light of day [105]. The ghost-written reversal on vitamin D dental caries prophylaxis did become published in JADA after some edits by the ADA CDT. It is unclear if the ADA CDT would have authorized the reversal on vitamin D dental caries prophylaxis had they been informed that the AMA Council on Nutrition (during this conflict) had decided to officially accept the vitamin D dental caries prophylaxis claim. Internal records suggest the ADA Secretary of the ADA CDT kept this information from the ADA CDT members until after all votes for the publication of the reversal on vitamin D dental caries prophylaxis had been obtained [105,106].

### 6.3. On Public Health and Micronutrient Deficiencies

*“Experts are often called in, not to provide factual information or dispassionate analysis for the purpose of decision-making by responsible officials, but to give political cover for decisions already made and based on other considerations entirely.”*

2009—Thomas Sowell -American author, economist, political commentator, social theorist.

The ADA CDT knew that the ghost-written announcement of the reversal on vitamin D dental caries prophylaxis ended up on their scientific agenda because of a conflict on advertising policies with the AMA, and not because of recent vitamin D-related scientific evidence.

If the ADA CDT were to follow their adopted scientific rules, they would not have been able to reverse on vitamin D dental caries prophylaxis which they had endorsed for 15 years [36]. Even though the ADA CDT lead vitamin expert was opposed to such an endorsement (in part on grounds of scope of practice), he cited a medical journal article in the yearly publication entitled “Accepted Dental Remedies” which referred to the public health significance of dental caries and vitamin D: that dental caries and pediatric osteoporosis could be signs of vitamin D deficiencies, that pediatric osteoporosis was prevalent, that dental caries offered a targeted screening opportunity to treat and prevent pediatric osteoporosis and that dental caries offered a useful criterion to estimate the human vitamin D requirement. A separate publication provides detailed analyses on the lack of evidence to justify a reversal on vitamin D dental caries prophylaxis [36].

The 1945 ADA CDT reversal on the role of micronutrient deficiencies in dental disease etiology is a second pivotal 20th century event which is key from an evidence-based perspective. To repeat, the ADA CDT was a council instructed to make decisions based on a set of scientific rules, not opinion.

### 6.4. A Scientific Endorsement Which May Have Contributed to a Micronutrient-Deficient Age

*“I ghost-wrote such an article …”*

Donald A. Wallace—1947 Secretary of the ADA CDT and reporting on the practice of ghost-writing. He also reported how he wrote a long paper as a basis for the fluoride reversal. [82] He was the likely author of the vitamin D reversal announcement.

The ADA CDT ignored their scientific rules and authorized the ghost-written reversal on vitamin D dental caries prophylaxis. One might never have had insights into how the ADA CDT justified ignoring a preponderance of evidence were it not for a last-ditch effort to resolve the ADA-AMA conflict on vitamin D dental caries prophylaxis with an in-person discussion with the AMA Secretary of the Council on Foods and Nutrition. 

The discussions between the AMA representative and the ADA CDT show that the dental ADA CDT members (about 50% of ADA CDT members were dentists) misrepresented the vitamin D literature and did not appear to be familiar with how vitamin D dental caries prophylaxis claims had been advertised in JADA. A detailed analysis of vitamin D related claims raised by ADA CDT members is presented in the Supplementary Materials and contrasted with published evidence (Supplementary Materials, part S3).

Dental ADA CDT members cited investigators who allegedly “did not differentiate too well between (vitamin) A and D” who allegedly had shown that the effectiveness of vitamin D dental caries prophylaxis could be due to “physical properties of the oil and not of the vitamin D content”, who allegedly had claimed that vitamin D2 “did not decrease the incidence of caries” (Supplementary Materials part S3). These vitamin D investigators, cited by name by the ADA CDT, had however reported the opposite, they had separated vitamin D from vitamin A, shown that the vitamin D effect was independent of the oil effect [110], and had suggested that vitamin D2 “at first sight seem, indeed, to be better than those obtained in former tests with cod-liver oil” (vitamin D3) [111]. 

The very first ADA Seal of Acceptance in JADA was for an advertisement reporting how vitamin D2 was needed “for cases where a marked susceptibility to caries indicates a serious lack of vitamin D [112]. Subsequent advertisements in JADA further emphasized how vitamin D2 was needed “for children who showed a marked susceptibility to dental caries” (e.g., [112,113]). Most vitamin D dental caries prophylaxis advertisements in JADA were for vitamin D2. Yet, a dental ADA CDT member suggested vitamin D2 was ineffective.

**Figure 4:**
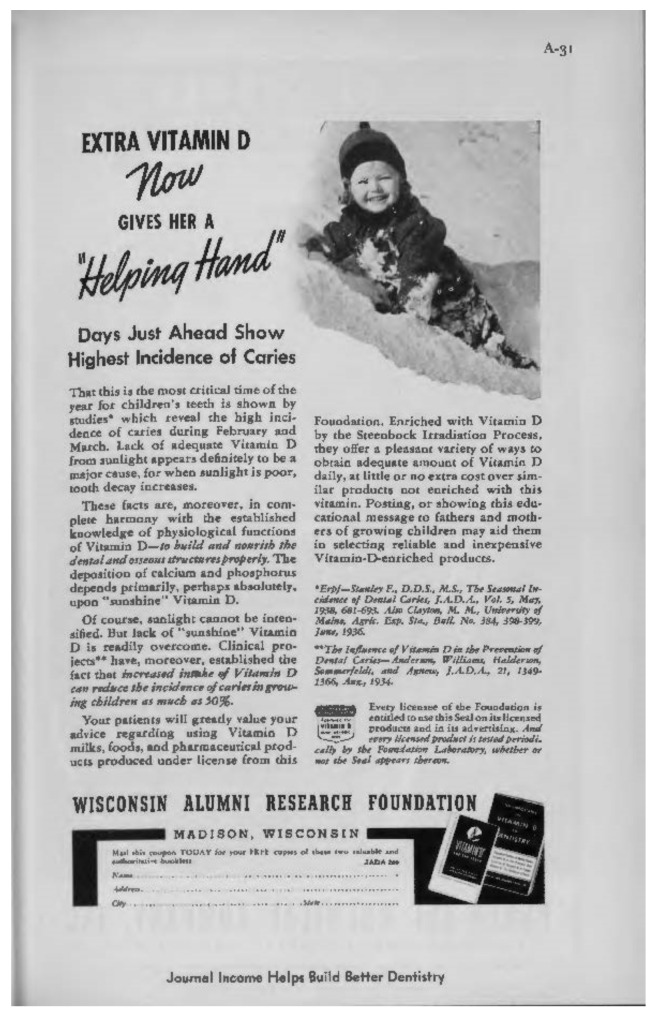
This advertisement appeared in JADA [114], suggested seasonality of dental caries, and provided a reference to a study supporting such a claim. Over 10 such seasonality studies had been conducted [115,116,117,118,119,120,121,122,123,124,125]. The National Research Council later concluded that seasonality of dental caries “merits further intensive investigation” [126]. Yet, a dental ADA CDT member reported how he had “never seen such evidence” [83].

Dental ADA CDT members appeared unaware of other scientific publications such as those related to the seasonality of dental caries (Figure 4). 

ADA CDT members misrepresented facts on other issues such as nutrition research in children, the AMA-described-vitamin D-sources, and the newly coined AMA dental caries prophylaxis claim (which the ADA CDT by now had been informed on) [112,113,127,128,129,130,131,132]. (See Supplementary Materials, part S3). 

That dental ADA CDT members misquoted vitamin D research is not unexpected. Dental research on the role of vitamin D was mostly conducted by physicians and scientists, not dentists. The diagnosis and treatment of nutritional disorders belonged to the medical scope of practice, not the dental scope of practice. It was outside of the scope of dental practice to perform the necessary medical examinations to diagnose and treat nutritional deficiencies. 

It was the AMA, not the ADA, who decided whether the daily recommended vitamin D intake should be increased by 50% for dental caries prevention. It was the AMA, not the ADA, which had a Council on Foods to assess vitamin D fortified food claims. It was the AMA Council on Foods, not the ADA CDT, which described the relationship between vitamin D and dental caries prevention as a “very important field” which the AMA was working on [88]. The ADA CDT was informed on these AMA deliberations on dental caries prevention with vitamin D. By 1946, this opinion that the dental scope of practice did not include diagnosing nutritional deficiencies was not a secret—the ADA CDT had published in JADA that where “it is practicable to do so, the patient’s physician should be consulted” when dental signs of nutritional deficiencies appear [35].

The ADA CDT had not worked on vitamin D scientific issues in the 15 years prior to the reversal. It should be noted here that the non-dental members (except for one) also showed no evidence of being familiar with the vitamin D dental caries literature which is not surprising. The ADA CDT members were recruited because of their specific areas of expertise. The ADA CDT had one vitamin and mineral expert—a biochemist—a person who was opposed to dentists prescribing vitamin D pharmaceuticals. The ADA CDT’s work consisted of approving those vitamin D brands which the AMA had approved, rejecting vitamin D brands which the AMA had rejected. These votes had been a monotonous affair; over 1000 votes over a 15-year period—almost always unanimous -with rarely a comment or a concern. 

The surprising part in the discussions of ADA CDT members with the AMA representative was not that the ADA CDT as a group (except for one ADA CDT member) appeared unfamiliar on vitamin D research, but that mostly the dental ADA CDT members misrepresented what vitamin D researchers had published. This could be described as wishful thinking on the part of some dental ADA CDT members, on what may have been pleasing for them to imagine. Misrepresenting what investigators have reported, wishful thinking, has been referred to as a pathological science -a term coined by the Nobel prize winner Langmuir to refer to the science of things that are not so [133].

In summary, it was wishful thinking which some dental ADA CDT members provided as evidence to justify their authorization of the reversal on vitamin D dental caries prophylaxis. This wishful thinking on the part of the ADA CDT was not motivated by aiming to protect ADA advertising revenues. In 1943, the chair of the ADA CDT (who was a dentist) said: “I would like to make this clear to (the ADA Board of Trustee member) that the ADA CDT has never been an advertising agent for the ADA and refuses so to be” …. “If we did assume that role (i.e., an advertising agent) we would lose our claim to any scientific integrity”. The actions of the ADA CDT during their first 15 years of existence frequently led to financial harm to the ADA and provide strong evidence that they were indeed not financially motivated in their decisions. Evidence is now presented that the dental members of the ADA CDT may have engaged in wishful thinking because of the ideological key which would make the dental profession equal to the medical profession. This ideological key—it is repeated here—was “dental science-knowledge” or dental “biological sciences” [69].

### 6.5. How Dental Ideology Can Become a Driver of Pathological Science

*“Ideology influences the scientific agenda and determines what to do with the discoveries” [134].*

2015—Yuval Noah Harari—professor Department of History at the Hebrew University of Jerusalem.

Several lines of evidence indicate that at least some dental members of the ADA CDT were motivated by an ideological scientific mindset. The Sugar Research Foundation had a scientific agenda which framed the research question within the context of finding an antidote to the harmful effects of sugar. An expert working for the Sugar Research Foundation reporting on internal research that sugar is harmful and that proposed antidotes are ineffective will probably be called a whistleblower and will likely not remain hired as an employee of the Sugar Research Foundation. Such an observation can be generalized to any organization. Expert clinicians—dentists in this case—working for professional organizations may be in no different situation– leading clinical experts at professional organizations will more likely be successful if they frame the research agenda to serve their profession—to support preventive and therapeutic approaches that fall within the dental scope of practice, to dismiss approaches that fall in the medical scope of practice. 

The medical management of dental diseases fell outside of the dental scope of practice. Vitamin D dental caries prophylaxis may have become collateral damage in the ADA-AMA conflict because some dental ADA CDT members re-framed the clinical research agenda to defend their scope of practice, to defend their scope of research. The primary medical vitamin D expert on dental caries at the AMA—a pediatrician named Jeans—reported how “dental research men” were in favor of the “oral environmental theory of dental caries”. What Jeans likely noticed was the “ideological key” providing the justification to separate the medical and dental profession. This ideological frame of mind among some dental ADA CDT members may have led to the pathological science statements needed to dismiss a preponderance of controlled clinical trial evidence. This ideological mindset created an impasse between the medical and dental profession. Jeans’ obituary describes him as an excellent mediator of conflicts and the most difficult task in his life was described as mediating the question on the cause of dental caries. It took over 10 years of “revisions after revisions” to finish the National Research Council’s report on dental caries (which he chaired). He died before this report was published [69]. 

The ADA ideological research agenda subsequently intensified thanks to—once again—political actions. The ADA Board of Trustees soon after 1947 decided that the ADA CDT—the prime scientific council within the ADA—should be limited to dentists. Since its creation in 1930, the ADA CDT had consisted of ~50% dentists, ~50% scientists or physicians. This panel had increased in size from 12 to 14 members in 1944 with the aim to expand the ADA CDT’s scope of authority to devices—a category which included toothbrushes. The ADA CDT however decided not to accept claims that toothbrushes prevented dental caries. Soon after this rejection, the ADA CDT became informed of plans to reduce their 14-member panel to 5 members who had to be dentists [82]. This aim largely materialized. In 1950, the ADA CDT consisted of 6 dentists.

A charter member of the ADA CDT had described the potential public health implications of this ADA Board of Trustees decision to restrict ADA CDT membership to dentists. He had reported how dental ADA CDT members could be counted on to serve their profession, how non-dental ADA CDT members (Ph.D. scientists or physicians) could be counted on to serve public health [108]. The ADA CDT stopped serving public health by 1950 if this ADA CDT charter member was correct in his assessment. 

Professional success within an organization correlates with supporting the ideological research agenda. The dental ADA CDT members whose ideology—it is suggested here—led to an inexplicable scientific reversal on vitamin D dental caries prophylaxis shared 7 presidential appointments at various national or international dental organizations and later appointments at the National Institute of Dental Research. The 1947 chair of the Caries Control Committee who helped defeat medical imperialism in “dental public health” guidelines was also a dentist and a president at 2 national dental associations, president of the International Association for Dental Research and first ADA vice-president. The JADA editor who had taken it on his own responsibility for the ADA to reverse on topical fluoride applications in the dental office, who had friendly relationships with the Sugar Research Foundation, was one of the first dentists to be elected a senior member to the prestigious Institute of Medicine (now the National Academy of Medicine). 

## 7. The Start of the Carbohydrate Age

*“In light of current laboratory and epidemiological research findings, the (American Dental) Association recognizes that it is neither advisable nor appropriate to eliminate from the American diet sugar-containing foods that provide necessary energy value for optimal nutrition” [135].*

1998—ADA Transactions.

The ADA initiated the pivotal reversal on the significance of carbohydrate restriction in 1947. The 1942 ADA national caries control program reported that “if patients can be taught that in the control of dental caries a reduction in the carbohydrate intake is necessary…toothbrushing would be beneficial largely from a social or cosmetic point of view” [21]. The 1949 ADA Dental Caries Control and Prevention program reversed on this public health advice and reported on the need for an “absolutely essential” [23] “well balanced diet” which “may not be accompanied by a low caries attack rate” and which does require toothbrushing, fluoride, and routine X-rays for the purpose of early dental caries intervention [24]. This represented a most fundamental reversal on the significance of dental diseases in relation to nutrition. Dental cavities reversed from being viewed as a marker for the chronic diseases of civilization to a marker which could be a reflection of a nutrition required for “proper growth and development” [24]. This fundamental reversal was made by the ADA Council on Dental Health which, unlike the ADA CDT, had made it a priority to serve dental public health, not public health. Serving “dental public health” is different from serving “public health”. To paraphrase the 1945 Belding’s prediction—making teeth “healthy” with fluoride so that poor nutrition can be ignored can “delay fundamental advances in public health” [45]. 

### On One of the First Public Health Endorsement of the Carbohydrate Age

*“When we came to the field of diet and nutrition, the Council felt that because the question of nutrition was so controversial we should leave it out completely” [136].*

1944—Leroy M.S. Miner—ADA president in 1936 and chair of the 1942 National Health Program Committee. He expressed a change of heart on the dental profession’s role in providing public health guidelines on nutrition.

The ADA Caries Control Committee and the ADA Research Committee, unlike the ADA CDT, did not have an adopted set of scientific rules. Committees operating on opinion can reverse on public health guidelines with a “we believe”. 

For instance, in 1942, the national caries control program had stated that a “toothbrush is not an effective instrument” in caries control. The executive secretary of the ADA Council on Dental Health reported in 1946 to the ADA CDT how there was “no real evidence” that toothbrushes prevented dental caries, how “from a public health point of view” a controlled trial was necessary “before we can tell people that the proper use of the toothbrush will reduce caries” [60]. Yet, the Caries Control Committee reversed in 1949 without such controlled trial evidence with the preface “It is now believed brushing of the teeth is of value” in caries control [24]. 

A brief review is presented on some of the opinions which may have led the ADA Caries Control Committee and the ADA Research Committee to reverse on the recommendation to restrict dietary carbohydrates. 

First, some opined that providing nutritional guidelines was too complex and too controversial. A physician who was a leading international nutrition authority and a NIH official informed the chair of the ADA Caries Control Committee that dental guidelines on nutrition must be in line with those issued by the government [42]. This advice meant that the Caries Control Committee had to cooperate with the National Academy of Sciences and at least 20 government agencies [42]. Implementation of a nutritional component in the Dental Caries Prevention and Control program at a public health level would furthermore require collaboration with all health groups including physicians, pediatricians, and with federal, state, and municipal health agencies, and lay groups [42]. 

Second, prescribing fluoride may have been opined to be more in line with the dental scope of practice than admonishing patients for having a sweet tooth. In the words of one dental educator: “Yes, dental caries can be controlled by limiting the amounts of starches and sweets consumed, but the method has little practical value, for very few people would be willing to make the necessary dietary concessions” [137]. Every dentist considering a lecture to patients about nutrition may have “the naturally occurring thought: Well, this is the last time we’ll ever see them” [138]. 

Third, one person on the Caries Control Committee suggested in 1943 not to list the aim “to reduce sugar consumption by direct means” [42]. Instead, he suggested to promote a well-balanced diet which he assumed would lead to a reduction in carbohydrate intake without explicitly advising so [42]. Another reason he provided for using the term “well-balanced” nutrition was that it was “obviously undesirable as well as impossible to eliminate all carbohydrate foods from the diet”. The final version of the Dental Caries Control and Prevention program did recommend restriction of added sugars, not of intrinsic sugars (See Supplementary Materials, part S4). In the 20th century, this turned out to be a slippery slope towards the appearance of sugar-containing foods in ADA educational material. A year later, in 1950, controversy regarding approval of “ice cream” in the diet in dental health education material appeared in the minutes of the ADA Council on Dental Health [139].

Fourth, pathological science may have also influenced the ADA Research Committee which worked in association with the Caries Control Committee. Key fluoride experts as either members or consultants had expressed opinions that “fermentable carbohydrates do not initiate caries” [140], that “once the teeth were formed, nutrition had nothing to do with the incidence of caries” [59], and that “sugar is not a cause of dental caries” [54].

An orthodontist and member of the ADA Council on Dental Health captured in his own words the aforementioned ideological key which started to separate the medical and dental profession. He stated: “Carbohydrates cause dental caries by a chain or series of actions. Any method that will break that chain or series of actions will prevent dental caries” [42]. The dental profession could start focusing on the down-stream elements of this chain, those chain links which fell in the dental scope of practice and as a result focus on interventions such as fluoride, fissure sealants, oral hygiene, or dental fillings. This is a logical comment and serves the private interests of the dental profession. However, to repeat, it is this ideological “dental public health” approach which in 1945 had been predicted to potentially delay fundamental advances in “public health”.

## 8. Discussion

A dental organization may have been among the first to open the doors to the fluoridated carbohydrate age simply because sugar and cereal executives regarded dental caries as one of their most pressing concerns. Some of these same industries (examples of private interests) may have led medical professional organizations to similarly reverse—in an absence of trustworthy evidence—towards high-carbohydrate nutritional guidelines [141,142]. Medical professional organizations -in part due to interprofessional conflicts (another private interest)— furthermore began ignoring the evidence that dental symptoms were sensitive markers for micronutrient deficiencies. Fluoride-supplemented, possibly micronutrient-deficient, high-carbohydrate nutritional guidelines emerged. The main conclusion of this report is that private interests can provide explanations on the origins and persistence of key reversals in nutritional guidelines, feats which, at least for some reversals, appear challenging to explain from an evidence-based-medicine perspective.

Non-professional organizations followed. The National Research Council (the operating arm of the National Academies) reported in 1989 how there was no evidence that sugar was associated with any chronic diseases other than dental caries and how further research might still show that “the contributory role of carbohydrates in the pathogenesis of caries can be effectively offset by fluoride” [143]. The expert panel behind such public health advice had no set of adopted scientific rules. The advent of evidence-based medicine in 1992—the use of rules applied to evidence—failed to change key features of the fluoridated carbohydrate age. Leading organizations such as the USDA and the WHO decided to ignore the core-principles of evidence-based medicine [6,11]. As was predicted by one dental editor in 1945, the support for universal fluoride—which became synonymous with “dental public health” may have delayed “fundamental advances in public health” [45]. Switching off a primary alarm bell for sugar and starches (dental pain) by measures such as fluorides and dental sealants coincided with the subsequent onset of an obesity and diabetes epidemic. 

In a fluoridated carbohydrate world, the harms of sugars became underestimated, the effectiveness of some fluoride treatments became overestimated. The American Diabetes Association reported in 1994 how sucrose caused dental caries in the general population and how it was *likely* that it would affect people with diabetes similarly [144]. The message of the American Diabetes Association was that older children and teens could “be taught how to incorporate sucrose-containing foods into a meal plan” [144], and that it just might be that sugar causes dental cavities in a diabetic pediatric population in the same way it did in non-diabetics. 

The pendulum may have started to swing back towards recognizing the potential harms of sugars and starches. The World Health Organization now recommends restriction of added sugars [145,146]. Sugar and starches are again increasingly recognized as a potential cause of diabetes [147,148]. The key elements of the fluoridated carbohydrate age however remain in place to this day. High-carbohydrate diets continue to be recommended as healthy, and consequently universal fluoride recommendations remain in place. 

The effectiveness of some fluoride treatments in preventing dental caries remain overestimated. For instance, the recommendation by the United States Preventive Services Taskforce (USPSTF) that primary care clinicians apply fluoride varnish to all children 5 years and younger is puzzling as it is an intervention of marginal to no effectiveness [149,150,151]. Contrast the USPSTF’s clinical guideline with the 1945 New Hampshire Public Health Statement by the dental profession who felt “obligated to convey to the citizens” that “no baby was ever born crying for candy”, that “the cultivation of artificial appetites for candy should be avoided.” That even though fluoride may prove helpful, “it must be borne in mind that the fundamental cause of dental decay is sugar and starches” [152]. The USPSTF guideline in 2021 has the potential to cause harm by providing a false sense of security on the effectiveness of topical fluoride applications as an antidote to sugar in children less than 5 years old.

This report has both strengths and weaknesses. A first weakness of this report is the lack of a discussion on the elephant in the room—the economics of providing nutrition for the global population may be dependent on processed foods high in carbohydrate content. This private interest was known in the 1940s and has only intensified with a tripling of the world population since 1950. Fluoride is an effective agent to prevent dental caries in this real-world setting where high-carbohydrate diets may be unavoidable for many. No such private interests however justify ignoring the principles of evidence-based medicine or providing paternalistic and misleading public health advice. The evidence that starches may cause diabetes should not be ignored in public health messages even if starches was the only solution to avoid famine. Similarly, the evidence that fluoride is not needed for those who have an ability to avoid the high-carbohydrate diets should not continue to be ignored. Universal fluoride recommendations appeared inappropriate to the ADA CDT in 1947 and can be viewed as still inappropriate. The American Diabetes Association for instance now views low-carbohydrate diets as acceptable and it should at least be considered that individuals adopting such a diet may no longer need fluoride.

A second important weakness of this report is ignoring anchoring bias—experts may rely heavily on the assumption that the key reversals initiated by dental and medical professional organizations during the 20th century were correct, were driven by science and not by private interests. Generations of physicians and dentists were educated as if the fluoridated carbohydrate age was a public health milestone. Anchoring bias combined with a lack of adhering to (or knowledge of) the rules of evidence-based medicine may be partly responsible for the persistence of the fluoridated carbohydrate age.

Other weaknesses of this study include ignoring the heavy workload of the ADA CDT and how its’ members were most likely unaware of the historical significance of those few decisions they made which played a role in opening the doors to the carbohydrate age. Such is the experience of most people in the midst of pivotal historical reversals [153]. A final weakness mentioned here is that it is impossible to discuss all biases which may have affected the reversals. Strong personal beliefs, for instance, may have influenced some ADA CDT members. One ADA CDT member involved in the key reversals reported how “candy has a place in the daily diet”, how he saw no problems in allowing candy manufacturers to advertise claims that candy provided high caloric value, staved off fatigue, and contributed “to the joy of living” [105]. Such a personal bias may contribute to advocating fluoride for all.

Additionally, it needs to be re-emphasized that the ADA CDT was remarkably accurate in its key 20th century decisions on dental caries prevention methods which were within the dental scope of practice such as toothpaste, toothbrushes, oral rinses, and also topical fluoride applications in the dental office for high-risk populations. With respect to the last point, the issue raised in this report is that the ADA CDT reversed in an absence of evidence on safety—an inexplicable aspect of the reversal. The ADA CDT’s decision turned out to be pivotal from a public health perspective because the ADA Council on Dental Health almost immediately generalized the ADA CDT’s position to include all children, not just high-risk children [24]. The ADA CDT may have only gone awry when it decided on a dental caries prevention method which was outside of the dental scope of practice—the medical management of dental diseases. That such a decision which was marred by pathological science has become engrained as conventional wisdom provides support for the need to study the historical development of public health guidelines [12].

A known weakness of all historical analyses is that none of the sequence of events described in this report can be interpreted as causal. Finally, it could be considered both a strength and a weakness that few what-if scenarios were explored. What if the JADA editor had allowed normal scientific processes on fluorides to proceed at the ADA CDT? Such “what if” analyses are science fiction work, this report primarily aimed to focus on what happened, not as much on what might have happened. There appears little doubt that the ADA CDT was planning to reverse towards approving topical fluoride applications in the dental office for high-risk populations, but we will never know when and how the reversal on safety and the greater public good would have materialized.

Finally, it should be explicitly repeated here that many fluoride treatments are effective in reducing dental caries susceptibility. It should also be emphasized that this report in no way telegraphs the concept that the prevention of dental caries is not important. This report aimed to telegraph the opposite point of view: that primary dental caries prevention with a restricted carbohydrate intake has always been regarded as unequivocally effective, and that private interests may have contributed to the common current view that secondary prevention with fluoride should take precedence over primary prevention.

Scientific grading systems for obtaining trustworthy public health advice have improved, adoption of these grading systems by some leading organizations may have worsened. The unsuspecting healthy continue to be exposed to public health messages which have not been validated in rigorous pivotal trials (or worse, which are opposite of controlled trial results) [154]. The core-principles of evidence-based medicine continue to be ignored by some leading organizations [6,7,8]. Content experts continue to be a majority of members on many writing panels against the advice of the 2011 National Academies report [155]. Additionally, many professional organizations continue to claim that they can simultaneously serve public and private interests [136]. The chronic persistence and insistence of some leading organizations to ignore the lessons learned from past public health disasters suggests private interests continue to influence public health guidelines.

## Data Availability

Data sharing not applicable—no new data generated.

## Supplementary Materials

The following supporting information can be downloaded at: https://www.mdpi.com/article/10.3390/nu14204263/s1, Part S1: Internal document sources; Part S2: Board of Trustees and public health policies; Part S3: On the ADA CDT and wishful thinking; Part S4: 1947 ADA dietary guidelines and current WHO guidelines compared. References [156,157,158,159,160,161,162,163,164,165,166,167,168,169,170,171,172,173,174,175,176,177] are cited in Supplementary Materials.

## Abbreviations

Dental caries is a disease which leads to the destruction and loss of teeth, and which has dietary carbohydrates as a necessary cause. Commonly used synonyms include dental cavities and dental decay.

ADA	American Dental Association
ADA CDT	The Council on Dental Therapeutics of the American Dental Association. This Council had adopted a set of scientific rules to guide their decisions. The Council was instructed not to operate on opinion and adopted a motto to serve public welfare in 1944.
AMA	American Medical Association
JADA	Journal of the American Dental Association
JAMA	Journal of the American Medical Association
FDA	Food and Drug Administration
