# Supplementary material for: Private Interests and the Start of Fluoride-Supplemented High-Carbohydrate Nutritional Guidelines
Source: Nutrients. 2022 Oct 12;14(20):4263. doi: 10.3390/nu14204263 (PMC9610923; doi:10.3390/nu14204263)
Supplement: Supplementary file 1 [file nutrients-14-04263-s001.zip › nutrients-1913108-supplementary.pdf]

Part S1. : Internal document sources

A first source is the Bulletin of the Council on Dental Therapeutics at the American Dental Association. This bulletin was published yearly and was a compilation of all the work performed by the Council during a given year. Once a year, the council met in person and those meetings could be attended by representatives of the American Medical Association or the Food and Drug Administration, members of the ADA Board of Trustees, the editor of the Journal of the American Dental Association, or other ADA officials. A stenographer was present during these meetings providing a record as to whom was present and a verbatim record of the discussions. During the remained of the year, the council communicated with the Secretary of the Council. These communications became part of the bulletin and include submissions for products by industry requesting acceptance as a dental therapeutic, voting records on the decision whether to accept or reject a product and allowable advertising claims, written comments by council members, letters from a variety of stakeholders addressed to the council, discussion of scientific position statements by the Councils, decision on which experts to select, etc. The yearly bulletins are indexed and typically contain (scientific) rules as an index word.

A second source of internal documents relate to the ADA Council on Dental Health. These records are less complete and can contain index to the minutes of the Council, a verbatim record of the discussions of meetings, letters to the Council, and appendices.

A third source of internal documents are those from the Sugar Research Foundation which are available online (https://www.industrydocuments.ucsf.edu/food/).

Part S2. Board of Trustees and public health policies

David McLean expressed the following on the topic of the Board of Trustees being a policy forming body: "Let's discuss it briefly. First: The report points out that legally, (i.e., according to By-Laws) the Board of Trustees is not a policy forming body. All policy is to be formulated by the House of Delegates. The report recommends that the Administrative Authority of the Board be extended. In actual practice, the Board has been a policy forming body." "

"It has been generally recognized that the House is too large a body to adequately discuss major problems of policy. The Board, composed of 13 men instead of 300, often takes hours to boil them down, prepare them for the House. Moreover, the Board members have continuity of contact with A.D.A. policies over a period of years; the Board is a "rotating" body so set up that there are always some members of several years' experience. Probably an answer to this problem would be to have changes of existing policy before the Board in anticipation of recommendation to the House. Routine reports could go direct to the House except for their financial considerations. The House itself, however, should certainly be cut in in half. This would give small (one delegate) states greater representation in proportion to larger states; the balance of power would not be affected. Distant states, having fewer delegates to send to the annual meeting, might be able to pay their expenses; thus insure experienced state organization workers to sit in the House rather than novices as now is often the case [1]."

A more detailed discussion of whether the Board of Trustees has the power to make ADA policies can be found in the ADA Council on Dental Health minutes, October 14-18, 1944. Page 1-12 [2]. An examination that the official policy of the ADA fell into one of four classifications: (1) Actions and policies approved by the House of Delegates, (2) Actions and policies approved by the Board of Trustees, (3) Actions and policies approved by committees and councils and yet lacking evidence that these had every been approved by the House of Delegates or the Board of Trustees, and (4) policies growing out of statements of various officers of the association. According to their read of ADA bylaws, the ADA constitution and bylaws were clear; policy-making powers rests with the House of Delegates, not the Board of Trustees. One exception was the Journal of the American Dental Association. They were supposed to superintend JADA which may be of relevance to this story as it could imply that they approved the editorial which initiated the 1947 fluoride reversal.

Part S3: On the ADA CDT and wishful thinking

Table S1. Verifiable Facts raised by ADA CDT members which were inconsistent with published evidence and/or ADA policy.

ADA CDT member	Dentist	Highest Appointments	Alleged mistakes in AMA report	Published evidence suggestion falsehood
Wallace, DA	✓	Secretary: ADA CDT	AMA claimed "Ice cream will definitely control caries"	There was no mention of ice cream in the AMA advertisement under dispute [3]. This mistake was caused by confusing a 1943 advertisement by the *Young Ice Cream* company in the *Montgomery Advertiser* [4] with the 1944 advertisement by *Carnation* published in the *Journal of the American Medical Association*[5]. This mistake may have been further compounded by inferring from the advertisement that the *Young Ice Cream company* advertised ice cream for dental caries prevention[4]. But this was not the case. The Young Ice Cream company advertised vitamin D milk. [6]
Hill, TJ	✓	President: IADR, ACD Appointed: NIDR	Mellanby claimed Vitamin D2 is ineffective	Mellanby had reported the opposite: better results were obtained with viosterol (vitamin D2). While she was careful to provide non-causal explanations for the superiority of vitamin D2, the industry advertised (and the ADA endorsed) that vitamin D2 supplementation was more effective than vitamin D3 and indicated for those individuals particularly susceptible to dental decay [7–14]. The ADA had endorsed claims that vitamin D2 fortification of cod liver oil was indicated when "teeth showed a tendency to decay easily" [8–13]. Vitamin D2 was ADA accepted and advertised as early as 1931 [11] and became the most frequently advertised form of vitamin D in the *Journal of the American Dental Association* from 1936 [15].
	✓		Rosebury …concluded that it was the oil and not vitamin D that prevented dental caries	Rosebury concluded: [16] *Vitamin D induced a decrease in caries independent of oil.*
Ostrander, FD	✓	President: ADA	"I have never seen such evidence that dental caries varies with the amount of sun"	At least eleven publications published prior to the debate supported the seasonality of dental caries (none of which were authored or co-authored by Becks[17–27]). The *National Academy of Sciences* concluded based on six studies that "there may be an association between the season of the year and the dental caries incidence which"… "merits further intensive investigation" [22–27]. Three of these six publications were published in JADA. [20,22,24] JADA advertised the seasonality of dental caries frequently [28,29] [30–32]. The seasonality of dental caries was often headlined with catchphrases such as the "toothache months" [28] or "When Sunshine decreases, dental caries increase" [32]. The advertisements carrying these catch phrases provided scientific references to the seasonality of dental caries [22,33]..
	✓		Boyd's work was on diabetic children eating a high fat diet	Boyd had investigated the role of nutrition in dental caries incidence in orthopedic patients (not diabetics), children living at home without an indication of being diabetics (allowed to eat candy at mealtime), celiacs who were on a very high carbohydrate diet[34], and children supervised in a metabolic chamber[35]. Boyd's life's mission appeared to be to demonstrate that dietary carbohydrates did not cause dental caries as long as the diet contained all the essential factors [34,35]. Based on this research in non-diabetics he promoted sugary high-carbohydrate diets combined with all essential factors including vitamin D. In a *New York Times* interview in 1944 Boyd referred to sugar as – "a cheap form of energy"…:"making meals attractive" [36] and he stated that he had no objections to candies and cookies [36].

Lyons, H	✓	Dean: 19 years President: ADA, AAP, ACD, AADS Appointed NIDR	Becks criteria for "when caries started" were implausible.	There was no evidence that Becks reported on caries criteria in relation to the seasonality of dental caries.
	✓		*Becks' was the "only" dental researcher responsible for the "seasonality and dental caries claim":*	Published literature identified Hermann Becks as a prolific dental researcher who published one article in 1946 (after the debate) [37] on the seasonality of calcium concentrations in the saliva, not dental caries. Becks' work " did not disclose any seasonal influence on rate of flow, calcium or phosphorus content of resting saliva." [37] No publications were identified where Becks investigated the seasonality of dental caries and neither did Becks refer to any seasonality work he might have done in his *1946 Journal of Dental Research* report [37].
	✓		Seasonality "has long been disproved."	National Academy of Sciences concluded that seasonality "merits further intensive investigation". .
Dobbs,EC	✓	Chair	AMA claims vitamin D should be obtained from sun	The AMA publication, *New and Nonofficial Remedies*, did not state that one should obtain their vitamin D requirements from the sun. The one mention on sun and vitamin D in *New and Nonofficial Remedies* reported that manufacturers could not claim that vitamin D "may not have all the beneficial effects of sun exposure". [38]
	✓		Mellanby's … speaks of A and D almost interchangeably	Mellanby initiated a study in children to differentiate the effect of A & D on dental caries as soon as this became technically feasible in 1927. This was 18 years before Dobbs' assertion that the effect of vitamin D and vitamin A was not well differentiated in Mellanby's work. Mellanby concluded in 1928: "The facts observed show that arrest of decay is essentially due to the presence of vitamin D in the diet, and that vitamin A (the anti-infective vitamin) possibly plays no part in this arrest" 9. The ADA CDT had specifically rejected endorsing vitamin A ("evidence for its usefulness to dentists … is not available" [39], and the ADA CDT had rejected endorsing fish oils with high vitamin A and low vitamin D concentrations ("At present there are no marked indications for its (vitamin A) use as a dental therapeutic "[40].
			(Mellanby's) work was done without controls	Mellanby may have been the first investigator in dentistry to start controlled clinical trials in humans in 1921.
			Mellanby' s work was on puppies	Mellanby was fully aware of the limitations of animal research and stated: "For the crucial test, investigations must be transferred from animals to human beings" ([41], p. 9). Starting in 1921 Mellanby became primarily involved with human research: clinical trials and epidemiological studies in England and Lapland. [7,41–46]
Logan, M		Professor	*the AMA asserted that vitamin D supplementation' definitely' led to a freedom from decay*	AMA stated: "Claims should not state or imply that vitamin D is the only important factor in caries prevention and arrest". The investigators of vitamin D trials promised freedom of decay with adequate dietary vitamin D intake [47]. The National Research Council later reported: "it was never claimed that vitamin D deficiency was the only cause of dental caries but rather if a partial vitamin D deficiency existed, the addition of vitamin D to that diet would result in a decrease in the dental caries increment" [48] (p.463).

Table S2. The ADA CDT was asked to provide a scientific basis for disagreeing with the AMA therapeutic claim for the role of vitamin D supplementation to prevent dental caries post-infancy (see Figure 1A). The confidential report provided

one science sentence that was substantiated with four outdated scientific references which were unrelated to vitamin D supplementation post-infancy. Three of these four references supported a role for vitamin D in dental caries.

**First author**	**Publication**	How old was the evidence in 1944?	Primary comparison studied	Evidence on AMA advertised claim		Author's conclusion on the role of vitamin D in dental caries prevention
				Vitamin D milk	post-infancy vitamin D supplementation	
Mackay	1931	14	46 rickets<br>40 controls	No	No	Vitamin D "deficiency may be one among a variety of etiological factors".
Hess	1931	14	71 rickets<br>24 controls	No	No	Children who had been protected from rickets had developed less caries than those who had not been protected
Eliot	1934	11	236 rickets*<br>215 control	No	No	Dental caries too was found more often in children with a known history of rickets.
Shelling	1936	9	126 rickets<br>150 controls	No	No	Administration of vitamin D in early infancy does not protect against caries

*severe and moderate combined

Part S4 -1947 ADA dietary guidelines and current WHO guidelines compared

Just like the WHO now, the ADA recommended to decrease the consumption of foods to which sugar was added during processing such as sweets, syrups, jams and jellies, sweetened beverages, pies, cakes, cookies, and doughnuts. Just like the WHO now, the ADA did *not* recommend reducing sugars intrinsic to fruits or complex carbohydrates in cereals or starchy vegetables. Just like the WHO now, the ADA recommended a reduction in the consumption of sugar, not an elimination. The reversal was a slippery slope. In 2002, the National Institute of Health recommended candy as a healthy snack [49].

References

1. McLean, D.W. The Trustee's corner. *The Journal - Published by the Southern California State Dental Association* **1943**, *X*, 12,11-14.
2. A.D.A. Council on Dental Health Meeting, *Minutes*; ADA Archives, Chicago, IL, USA; 14-18 October1943
3. Anonymous. Children's teeth need vitamin D after infancy, too. *The Journal of the American Medical Association* **1944**, *124*, 48.
4. Anonymous. Yes! Scientific tests proved that one quart daily of Vitex D milk cut down tooth decay in children. *Montgomery Advertiser* Sunday, April 25 1943.
5. Anonymous. Children's teeth need vitamin D after infancy, too. *J. Am. Med. Assoc.* **1944**, *124*, 48.
6. American Dental Association. Council on Dental Therapeutics. Bulletin of the Council on Dental Therapeutics. **1943**, 280.
7. Mellanby, M.; Pattison, C.L. The Action Of Vitamin D In Preventing The Spread And Promoting The Arrest Of Caries In Children. *The British Medical Journal* **1928**, *2*, 1079-1082.
8. Anonymous. Lack of Vitamin D, probably the most important factor in producing poor teeth. *J. Am. Dent. Assoc.* **1930**, *17*, A-10.
9. Anonymous. Teeth develop slowly, eruption is delayed when the diet supplies too little vitamin D... *J. Am. Dent. Assoc.* **1930**, *17*, A-10.
10. Anonymous. Help them build sound, strong, even teeth with Bottled Sunshine. *J. Am. Dent. Assoc.* **1931**, *17*, A-10.
11. Anonymous. Help them build sound, strong, even teeth with Bottled Sunshine. *J. Am. Dent. Assoc.* **1931**, *18*, A-10.
12. Anonymous. More than one way to help them build a fine set of teeth. *J. Am. Dent. Assoc.* **1931**, *18*, A-10.
13. Anonymous. Protect their first set of teeth with Bottled Sunshine. *J. Am. Dent. Assoc.* **1931**, *18*, A-10.
14. Anonymous. Well-spaced, sound, straight teeth are built by Bottled Sunshine. *J. Am. Dent. Assoc.* **1931**, *18*, A-10.
15. Anonymous. "How does vitamin D help build and protect strong, even teeth, Mr. Dentist?". *J. Am. Dent. Assoc.* **1936**, *23*, A52.
16. Rosebury, T.; Karshan, M. Susceptiblity to Dental Caries in the Rat: VIII. Further Studies of the influence of Vitamin D and of Fats and Fatty Oils. *J. Dent. Res.* **1939**, *18*, 189-202.
17. East, B.R. Nutrition and Dental Caries. *Am. J. Public Health Nations Health* **1938**, *28*, 72-76.
18. East, B.R. Mean Annual Hours of Sunshine and the Incidence of Dental Caries. *Am. J. Public Health Nations Health* **1939**, *29*, 777-780.
19. East, B.R.; Kaiser, H. Relation of Dental Caries in Rural Children to Sex, Age, and Environment. *Am. J. Dis. Children* **1940**, *60*, 1289.
20. Kaiser, H.; East, B.R. Association of dental caries in children with sex, age, and environment. *J.A.D.A.* **1940**, *27*, 1430.
21. Franci, G.B. Ulteriore contributo allo studio della etiogenesi della carie dentale. Diffusione della carie dentale nei bambini dell scuolo di Tripoli d'Africa. *Atti R. Accad. Fisiocrit. Siena* **1934**, *2*, 609.
22. Erpf, S.F. Dental caries and paradental distubances. The seasonal incidence of dental caries. *J. Am. Dent. Assoc.* **1938**, *25*, 681.
23. McBeath, E.C.; Zucker, T.F. The Role of Vitamin D in the Control of Dental Caries in Children. *The Journal of Nutrition* **1938**, *15*, 547-564.
24. Price, W.A. Seasonal variation in butterfat vitamins and their relationship to seasonal morbidity, including dental caries and disturbed calcification. *J. Am. Dent. Assoc.* **1930**, *17*, 850.
25. Lathrop, B.M. Seasonal variations in the incidence of dental caries. A preliminary report. *Am. J. Mental Deficiency* **1941**, *46*, 33.
26. Lathrop, B.M. Seasonal variation in the incidence of dental caries. *Am. J. Mental Deficiency* **1943**, *47*, 260.
27. Staff Members, Ellen H. Richards Institute Relationship of season of year to nutritional status. *Chemistry Leaflet* **1942**,*15*, 70.
28. Anonymous. With "toothache months" ahead. *J. Am. Dent. Assoc.* **1939**, *26*, A-40.
29. Anonymous. Now is the Time they Need Extra Vitamin D to Protect Their Teeth. *J. Am. Dent. Assoc.* **1939**, *26*, A31.
30. Anonymous. Extra Vitamin D Now gives her a "Helping Hand" Days Just Ahead Show the Highest Incidence of Caries. *J. Am. Dent. Assoc.* **1940**, *27*, A-31.
31. Anonymous. Vitamin "D" can help them keep the Sunshine of their Smiles. *J. Am. Dent. Assoc.* **1940**, *27*, A24.
32. Anonymous. When Sunshine decreases, dental caries increase Vitamin D milk will help protect your patient's teeth in the "Sun-Poor" months. *J. Am. Dent. Assoc.* **1943**, *30*, A38.
33. Clayton, M.M. *Annual Report of the Maine Agricultural Experiment Station, Bulletin* **1936**, *384*, 398-399.
34. Boyd, J.D.; Drain, C.L.; Nelson, M.V. DIETARY CONTROL OF DENTAL CARIES. *Am. J. Dis. Child.* **1929**, *38*, 721.
35. Boyd, J.D.; Drain, C.L.; Stearns, G. Nature of Diet and its Relationship to Dental Caries. *Proc. Soc. Exptl. Biol. and Med.* **1937**, *36*, 645.
36. Holt, J. News of Food: Iowa State Pediatrician Lists Five Practices That Lead to Poor Nutrition of the Young. *New York Times* May 27, 1944 1944, p. 18.
37. Wainwright, W.W.; Becks, H. Human Saliva: XVIII. Is there a Seasonal Influence on Rate of Flow and Calcium and Phosphorus Content of Resting Saliva? *J. Dent. Res.* **1946**, *25*, 285-291.
38. Council on Pharmacy and Chemistry. New and non-official remedies. *American Medical Association* **1945**, 602.
39. American Dental Association. Council on Dental Therapeutics. Bulletin of the Council on Dental Therapeutics. **1933**, 158.
40. American Dental Association. Council on Dental Therapeutics. Bulletin of the Council on Dental Therapeutics. **1932**, 510.
41. Mellanby, M. *Diet and the teeth: an experimental study*; H. M. Stationery off.: London,, 1929; p. 3 v.
42. Mellanby, M.; Pattison, C.L. Some factors of diet influencing the spread of caries in children. *Br. Dent. J.* **1926**, *XLVII*, 1045-1057.
43. Mellanby, M.; Pattison, C.L. Remarks On The Influence Of A Cereal-Free Diet Rich In Vitamin D And Calcium On Dental Caries In Children. *The British Medical Journal* **1932**, *1*, 507-510.
44. Mellanby, H. Dental Hypoplasia And Caries Among The Children Of Finnish Lapps. *The British Medical Journal* **1940**, *1*, 682-686.

45. Mellanby, M.; Coumoulos, H. The Improved Dentition Of 5-Year-Old London School-Children: A Comparison Between 1943 And 1929. *The British Medical Journal* **1944**, *1*, 837-840.
46. Medical Research Council - Committee on dental disease. *The influence of diet on caries in children's teeth (final report)*; H.M. Stationery off: London, 1936; p. 10.
47. Hujoel, P.P. Vitamin D and dental caries in controlled clinical trials: systematic review and meta-analysis. *Nutr. Rev.* **2013**, *71*, 88-97, doi:10.1111/j.1753-4887.2012.00544.x.
48. National Research Council (U.S.). Committee on Dental Health; Toverud, G.; National Academy of Sciences (U.S.). *A survey of the literature of dental caries*; National Academy of Sciences, National Research Council: Washington, 1952; pp. ix, 567 p.
49. National Cholesterol Education Program. Third Report of the National Cholesterol Education Program (NCEP) Expert Panel on Detection, Evaluation, and Treatment of High Blood Cholesterol in Adults (Adult Treatment Panel III) final report. Circulation 2002, 106, 3143-3421.
